# Characterising ChIP-seq binding patterns by model-based peak shape deconvolution

**DOI:** 10.1186/1471-2164-14-834

**Published:** 2013-11-26

**Authors:** Marco-Antonio Mendoza-Parra, Malgorzata Nowicka, Wouter Van Gool, Hinrich Gronemeyer

**Affiliations:** Equipe Labellisée Ligue Contre le Cancer, Department of Functional Genomics and Cancer, Institut de Génétique et de Biologie Moléculaire et Cellulaire (IGBMC)/CNRS/INSERM/Université de Strasbourg, BP 10142, Illkirch Cedex, 67404 France; Institute of Molecular Life Sciences, University of Zurich, Winterthurer Strasse 190, Zurich, CH-8057 Switzerland

**Keywords:** ChIP-seq, Quality control, Next-generation sequencing, Massive parallel sequencing

## Abstract

**Background:**

Chromatin immunoprecipitation combined with massive parallel sequencing (ChIP-seq) is widely used to study protein-chromatin interactions or chromatin modifications at genome-wide level. Sequence reads that accumulate locally at the genome (peaks) reveal loci of selectively modified chromatin or specific sites of chromatin-binding factors. Computational approaches (peak callers) have been developed to identify the global pattern of these sites, most of which assess the deviation from background by applying distribution statistics.

**Results:**

We have implemented MeDiChISeq, a regression-based approach, which - by following a learning process - defines a representative binding pattern from the investigated ChIP-seq dataset. Using this model MeDiChISeq identifies significant genome-wide patterns of chromatin-bound factors or chromatin modification. MeDiChISeq has been validated for various publicly available ChIP-seq datasets and extensively compared with other peak callers.

**Conclusions:**

MeDiChI-Seq has a high resolution when identifying binding events, a high degree of peak-assessment reproducibility in biological replicates, a low level of false calls and a high true discovery rate when evaluated in the context of gold-standard benchmark datasets. Importantly, this approach can be applied not only to ‘sharp’ binding patterns - like those retrieved for transcription factors (TFs) - but also to the broad binding patterns seen for several histone modifications. Notably, we show that at high sequencing depths, MeDiChISeq outperforms other algorithms due to its powerful peak shape recognition capacity which facilitates discerning significant binding events from spurious background enrichment patterns that are enhanced with increased sequencing depths.

**Electronic supplementary material:**

The online version of this article (doi:10.1186/1471-2164-14-834) contains supplementary material, which is available to authorized users.

## Background

Chromatin immunoprecipitation (ChIP) combined with high throughput sequencing is widely used for characterizing the genome-wide association pattern of chromatin-interacting factors and histone or DNA modifications, for which selective tools for affinity purification, mostly antibodies, exist. While ChIPed DNA was first analysed at genome-wide level by hybridization to genomic tiling arrays (also known as ChIP-on-chip or ChIP-chip), direct sequencing is generally used these days (referred to as ChIP-seq). Massive parallel sequencing has overcome several limitations of the array-based (ChIP-chip) approach; such as spatial resolution, signal-to-noise ratio, dye and the probe-dependent hybridization biases and costs (for a detailed comparison of the two approaches see [1]); thus ChIP-seq is becoming the method of choice for mapping protein-chromatin interactions and chromatin modifications at global level.

Irrespective of whether ChIP-chip or ChIP-seq is used, the aim of the corresponding data analysis is to identify patterns in the reconstructed signal profiles that reflect the *bona fide* enrichment of the factor/modification of interest across the entire genome. Several pattern reconstruction methodologies have been described to date using approaches based on different concepts to define what constitutes an enrichment event or peak. The simplest concept defines an enrichment region based on a user-chosen read count intensity threshold [2, 3]. Other methodologies evaluate background levels from control (non-enriched) datasets to assess enrichment confidence p-values in the chromatin immuno-precipitated (ChIP) profile from a binomial distribution model [4, 5]. In the same manner, when no control samples are available, the background is usually estimated from a Poisson distribution model [4, 6, 7].

In the last years another group of peak callers was developed which use the signal enrichment dependency in a spatial context to discover significant binding events [8–11]. Importantly, this new family of peak callers defines significant binding events from the consecutive behaviour of enriched and non-enriched regions by applying Hidden Markov models (HMM), thus assessing its significance from enrichment properties rather than describing only differences relative to the background.

Finally, a new generation of peak callers exploits the properties of expected binding patterns. Among them, PeakRanger complements the use of the background modelling by using in a second round a “summit-valley-alternator” algorithm to scan for significant summits [12]. Others assess the shape of the observed binding patterns either by applying topological tree-based statistics [13], or by elucidating properties of the forms associated to the enrichment profiles [14].

Here we introduce MeDiChISeq, a model-based deconvolution approach, originally developed to evaluate ChIP-chip profiles [15]. Importantly, MeDiChISeq takes advantage of the shape of the binding event itself as a resource for identifying them in an accurate manner; thus by providing a higher power of discrimination between true binding events and artifactual read-count enrichment patterns. MeDiChISeq computes a model from a selected subset of the multiple binding events that constitute a genome-wide profile; then, this model is used as a deconvolution kernel to predict global binding/modification events, which are further validated by applying a non-parametric bootstrapping approach. The performance of MeDiChISeq has been compared with various other peak callers that are representative of the different approaches currently used to define significant binding events in ChIP-seq profiles.

## Implementation

MeDiChISeq is based on MeDiChI, a model-based deconvolution method designed for the analysis of ChIP-chip datasets [15] to which an important number of novel implementations have been added to enable the analysis of datasets generated by massive-parallel sequencing. Specifically, while the regression-based calculator embedded on MeDiChI is essentially the same in MeDiChISeq (see Additional file 1), the major novel implementations incorporated for transforming MeDiChI into a Peak caller dedicated to the analysis of ChIP-Seq datasets comprise (1) the preprocessing of mapped sequence files to generate read-count intensity files compatible with MeDiChI readout; (2) the enhancement of the peaks’ confidence assessment by including local and global background comparisons as well as the use of input control datasets when available and (3) the implementation of a multicore processing structure to accommodate computation requirements observed when MeDiChI was applied to larger genomes than those that have been used for its release. These novel implementations are described below in more detail.

### ChIP-seq datasets

MeDiChISeq processes mapped sequence files in different formats (e.g. BED, BAM). Read-count intensity profiles are reconstructed from mapped read files by elongating each read to a user-defined length (default read elongation: 150nt) and counting the elongated read overlaps within a defined window (default wiggle-format files resolution: 10nt). While the read elongation parameter is generally provided by the user, we have incorporated in MeDiChISeq a function that predicts a suitable read elongation from the information retrieved in the ChIP-seq profile itself (see Additional file 2 and below).

In ChIP-chip the reconstructed signal intensity is generated by comparing the immunoprecipitated information (IP) with the control dataset, while in ChIP-seq the IP and control datasets are processed separately. Therefore, MeDiChISeq takes as an additional file (when available) a control dataset for improving the confidence assessment of the identified binding events (see below).

### Establishing a representative binding pattern by applying an iterative learning process

One of the main advantages of MeDiChISeq is its capacity of inferring a representative binding pattern (referred to as “kernel”) from the provided ChIP-Seq dataset. As illustrated in the Additional file 3, this is performed by fitting a binding pattern model to a reduced number of genomic regions, which are selected by applying a read-count intensity cutoff criterion. This cutoff can be defined as a given read-count intensity or by a quantile intensity parameter.

Model fitting is performed in an iterative manner by evaluating in each round the number of peaks that fit the best to the current model and adjusting its parameters (shape and scale of Gamma distribution) by minimizing the regression residuals. The formalism of this procedure is extensively described in [15] and its implementation for ChIP-seq datasets is detailed in the Additional file 1.

### Sequenced reads elongation parameter inferred from ChIP-seq strand-specific information

In ChIP-seq assays the reconstruction of factor binding/chromatin modification profiles is currently performed by applying a computational elongation of the sequenced reads prior read-count intensities assessment. This elongation step is performed because each sequenced read corresponds to the 5’-ends of the fragmented immunoprecipitated chromatin. Importantly, the applied elongation distance, which corresponds generally to the fragmented chromatin length, is important for proper assignment of a factor binding site or chromatin modification.

While the read elongation parameter is generally provided by the user (based on the experimental conditions), we have incorporated in MeDiChISeq a function that could predict a suitable read elongation from the information retrieved in the ChIP-seq profile itself. In fact, while previous studies assessed the read elongation distance by evaluating the distance between the forward and reverse enriched reads [7], MeDiChISeq applies the iterative linear regression model fitting in a strand-specific manner without read elongation. This preliminary step infers the DNA fragment length per strand (ideally both strand-specific fragment lengths are the same); subsequently these are combined to define the read elongation parameter (see Additional file 2).

### Genome-wide identification of significant binding events by using the modeled representative binding pattern (kernel)

Binding site identification by MeDiChISeq is based on the assumption that all binding patterns associated to a given immunoprecipitation assay might present similar peak shape characteristics. Thus, the representative binding pattern or kernel modeled by the iterative regression approach is used to deconvolve binding events over the entire ChIP-seq profile. For this, the dataset is subdivided into overlapping windows (default parameter 20,000 nt window length; a contiguous window overlap is defined to cover at least one peak length) and the pre-computed kernel is used to identify those enrichment patterns that fit best.

Like in the case of MeDiChI, the likelihood of an enrichment event to match the trained kernel is related to the ChIP-seq background and is estimated by applying a non-parametric bootstrap approach [15]. MeDiChISeq compares for this purpose the putative binding sites identified by kernel fitting with the “kernel-fitting residuals” (i.e., those not complying with the model, and corresponding to the background). Moreover, these residuals are further deconvolved to identify potential patterns that would match with the operative kernel despite their possible background characteristics. Finally, each putative binding site is compared with its surrounding background in a local (default size of this centered surrounding windows is 5,000; 10,000 and 15,000nt) and global (genome-wide background) context.

The use of three different window sizes facilitates classifying the surrounding of potential binding sites as background. MeDiChISeq provides to each identified binding site local confidence p-values for all three evaluated windows and a global p-value. To provide an overall confidence estimate based on both global and local p-values, these descriptors were combined into a single confidence indicator (Fisher’s combined probability test).When available, a control dataset (e.g., non-enriched sample or IP with non-specific IgG) is included during the binding site assessment. Indeed, whenever an enrichment event matches with the trained kernel, the kernel-fitting process is also performed in the control dataset for the corresponding genomic region. If in a given chromatin region both the enrichment and the control dataset comply with the trained kernel, the confidence of the identified binding site in the immunoprecipitated dataset is corrected as follows:

This approach enhances the confidence of the predicted binding event by evaluating its uncertainty from different perspectives, namely relative to a local background, relative to the identified patterns across the genome (global background) and relative to the enrichment seen in the control sample. Note that the described control sample-based confidence correction is based on the assumption that the compared datasets (IP and control) present comparable sequencing depth levels. It is important to mention that some methodologies apply in case of divergent sequencing depths linear scaling corrections; however we have shown in a previous study that important differences in sequencing depths may give rise to non-linear differences between compared datasets [16].

In contrast to other methods, we do not suggest a default p-value threshold but provide a comprehensive list of all identified binding sites (complying with the kernel fitting) and their associated confidence descriptors such that the user can chose the optimal confidence threshold. In fact, defining a default p-value threshold may be misleading for inexperienced users, who may consider such reference as a gold standard rather than evaluating by other approaches the degree of false calls for a given p-value. Instead, we propose a graphical approach for estimating p-value levels, which may preferentially be associated to background behavior (described in the MeDiChISeq vignette; Additional file 4).

### MeDiChISeq implementation and performance

MeDiChISeq has been implemented in R and is designed to operate by multicore processing to accommodate computation requirements during linear regression fitting and bootstrapping.

For users who are interested in evaluating sites identified by another peak caller, MeDiChISeq offers an option in which only defined regions can be deconvolved. The R package, vignette and manual of MeDiChISeq are available as additional files (Additional files 4, 5 and 6). Note that these files can also be downloaded from our homepage http://igbmc.fr/Gronemeyer_MeDiChISeq.

## Results and discussion

**Figure 1:**
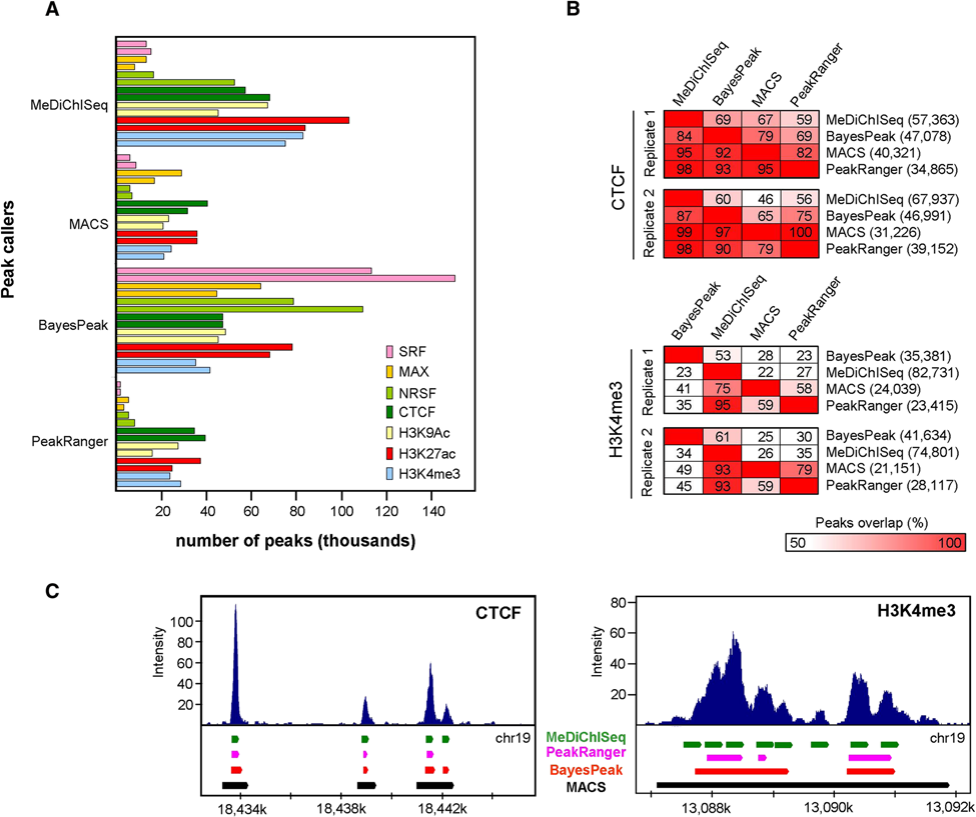
**MeDiChISeq performance evaluated in the context of several ChIP-seq datasets and relevant Peak calling algorithms. (A)** MeDiChISeq and three other peak callers (MACS, BayesPeak and PeakRanger) were used to identify binding events in ChIP-seq datasets for three TFs (SRF, MAX, NRSF), the sequence-specific insulator protein CTCF and two histone modification marks (H3K9Ac, H3K27Ac, H3K4me3). The default confidence threshold parameters described for each peak caller were applied to assess the number of peaks per dataset. Note that for each ChIP-seq two biological replicates were processed. **(B)** Peaks commonly identified by two of the indicated peak callers for two replicates of CTCF (top panel) and H3K4me3 (bottom panel) are displayed as percentages of the total sites found by a given method (indicated at the right). **(C)** Representative genome browser screenshots illustrating the ability to deconvolve binding/modification patterns of peak callers. Note that most of the peak callers identify a similar number of “sharp” binding events for CTCF, while MeDiChISeq has the highest potential of deconvolution for the H3K4me3 pattern.

In this study the performance of MeDiChISeq has been evaluated with a large number of publicly available ChIP-seq datasets. These include the TFs SRF, MAX, NRSF [17] and the sequence-specific insulator protein CTCF [18], all of which present sharp peaks in their ChIP-seq profiles. Moreover, also broad patterns characteristic of some histone modifications, such as histone H3 lysine 4 trimethylation (H3K4me3), and lysine 9 (H3K9Ac) or lysine 27 (H3K27Ac) acetylation, were also included using published data sets [18]. Importantly, MeDiChISeq performance was compared to three other peak callers, which are representatives of the different methodologies implemented over the years: MACS models the background according to a Poisson distribution [7], BayesPeak takes advantage of a fully Bayesian hidden Markov model to identify binding events [10], and PeakRanger applies in addition to background modelling, in a second round a “summit-valley-alternator” algorithm to scan for significant summits [12]. The relevant parameters in which each peak caller has been used are provided in the Additional file 7. As illustrated in Figure 1A, all four peak callers predict a variable number of significant peaks when default confidence threshold conditions are used (MACS: p-value < 1×10^-5^; BayesPeak: posterior probability or PP > 0.5; PeakRanger: p-value < 1×10^-4^, FDR < 0.01; MeDiChISeq: no confidence cutoff; instead the number of peaks complying with the kernel are given) suggesting *a priori* that default parameters may have to be optimized for comparative studies (see also [19]). In general, we observed that PeakRanger and MACS display a more conservative behaviour than MeDiChISeq and BayesPeak when comparing the total number of predicted peaks. Note that the number of MeDiChISeq sites corresponds to those complying with the kernel fitting and have not been filtered by any other threshold criterion. Even more importantly, differences in the number of sites identified by each peak caller are observed also with biological replicates, which likely reflect inherent differences in the characteristics of each of such datasets. Note that in the present study we considered only ChIP-seq profiles that were published as biological replicates.

To compare commonly identified sites we used the predicted peak summits ±50nt flanking sequences; as BayesPeak does not specify summits, the centre of the predicted peak base was used. This comparison revealed that MeDiChISeq identified the majority of sites predicted by the other methods (Figure 1B, Additional file 8). Notably, when comparing the fraction of peaks shared among peak callers MeDiChISeq performs best for both sharp and broad binding patterns (CTCF and H3K4me3), while most of the other peak callers present significantly lower fractions of shared peaks, as seen for H3K4me3 (Figure 1B). This observation correlates with the high number of MeDiChISeq-identified sites relative to the other peak callers resulting from the efficient deconvolution by MeDiChISeq. In fact, as illustrated in Figure 1C, MeDiChISeq annotated 8 distinct loci of H3K4me3 chromatin modifications, where the other peak callers identified one, two or three sites. We noted that these differences in the deconvolution potential of peak callers were less pronounced for sharp binding patterns (Figure 1C, left panel).

### MeDiChISeq’s sensitivity evaluated by their performance in reproducibility assays

Figure 1 illustrates that a comparison of peak caller performances under default parameters is unsatisfactory. In fact, default confidence thresholds that are too relaxed will increase the amount of false positive calls, while too stringent conditions will produce false negatives. To circumvent this problem, peak caller performance can be evaluated in the context of reproducibility assays by comparing binding site predictions for biological replicates and ranking them according to confidence descriptors. The underlying assumption is that true binding sites will be retrieved in both replicate datasets within a similar confidence ranking, while low confidence peaks, which are expected to contain also false positives, will show lower consistency in the reproducibility assay. The consistency between ranked peak confidence descriptors was previously formalized based on a copula mixture model, which estimates the probability that each pair of peaks is reproducible. This probability was described as “Irreproducibility Discovery Rate (IDR) [20] and has been used by the ENCODE consortium to identify a transition from signal to noise when peak caller binding site predictions were evaluated [21].

**Figure 2:**
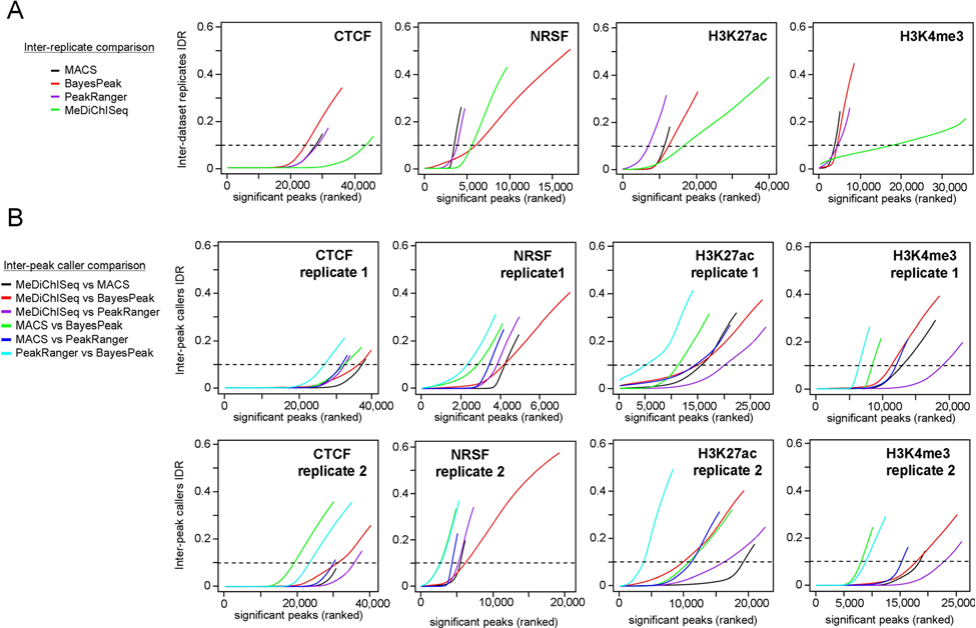
**Irreproducibility Discovery Rate (IDR) assays to compare peak calling algorithms. (A)** IDR assay comparing biological replicate datasets (see text for details). Note that for H3K4me3, MeDiChISeq continues to find significant common events in compared replicate datasets with slowly increasing IDR while the IDRs sharply increase for the three other peak callers around 5,000 significant peaks commonly identified in the replicates. **(B)** Similar reproducibility analysis but performed by pairwise comparison of binding site predictions by the different peak callers indicated at the left (“virtual replicates”). This approach reflects the concordance in binding site identification between two peak callers. Note that in all illustrated IDR assays, MeDiChISeq predictions have the lowest IDR levels for the highest number of significant binding sites. In **(A)** and **(B)** dashed lines indicate IDR levels of 0.1; i.e. a reproducibility level of 90%.

Here we have compared peak caller performance in the context of reproducibility across replicate ChIP-seq datasets. Importantly, MeDiChISeq showed the highest number of reproducible peaks in CTCF and NRSF ChIP-seq datasets (Figure 2A). Also for broader patterns like H3K27ac and H3K4me3 MeDiChISeq identified the highest number of reproducible peaks at acceptable IDR thresholds (e.g., 0.1 or 90% reproducible discovery). Note that the IDR progression curve for the histone modification mark H3K4me3 continues to increase rather slowly above this threshold, suggesting retrieval of an important number of irreproducible events among the significant top-ranked peaks in the replicate dataset. That the other peak callers identify less than 5,000 H3K4me3 peaks with IDR levels below 10% supports the view that for broader binding patterns the assessment of IDRs by applying standard approaches becomes suboptimal [20].

An important limitation of the above analysis is the potential variability between compared biological replicate datasets, as technical differences between the compared profiles may exist (e.g., sequencing depth differences; Peak caller deconvolution performance for broad patterns). To circumvent this limitation, we treated the predictions of two peak callers as “virtual replicates” for IDR analyses for a number of individual ChIP-seq datasets (Figure 2B). We thus ask if two peak callers identify binding events/marks in the same ChIP-seq dataset with similar confidence (i.e., if a top ranked peak of peak caller A is also top ranked by peak caller B). This novel type of comparison demonstrated that MeDiChISeq identifies higher numbers of reproducible peaks when compared with other methods. In fact, in the case of CTCF datasets, MeDiChISeq-MACS performed best for the first replicate, while MeDiChISeq-PeakRanger won in the case of the second replicate (Figure 2B). Importantly, evaluation of H3K27ac and H3K4me3 ChIP-seq datasets by this approach revealed large differences in reproducibility performance of the peak callers. PeakRanger and BayesPeak systematically performed worst, while MeDiChISeq versus any other peak caller gave the best scores in either biological replicate. Note that the particular IDR patterns observed for H3K4me3 in an inter-replicate comparison (right panel in Figure 2A) was not seen when the inter-peak caller performance for each replicate dataset was compared (right panels in Figure 2B), suggesting that it results from significant divergence between the “biological replicate datasets”. Overall these analyses showed that MeDiChISeq systematically identified the most reproducible events among biological replicates and peak caller annotations, thereby revealing the high sensitivity and reliability of this approach.

### MeDiChISeq’s specificity in the context of curated benchmark datasets

In addition to identifying the highest number of true binding events (sensitivity), a good peak caller algorithm is expected to produce the lowest amounts of false positives (specificity). As indicated above, IDR studies are expected to identify a transition from signal to noise when evaluating peak callers’ binding sites reproducibility. In this manner, the highest number of significant binding sites at the lowest IDR, as observed in the case of MeDiChISeq performance (Figure 2), reflects a high degree of sensitivity and specificity, at least in the context of reproducible binding site discovery in biological replicates or when comparing different peak callers’ performance per ChIP-seq dataset.

**Figure 3:**
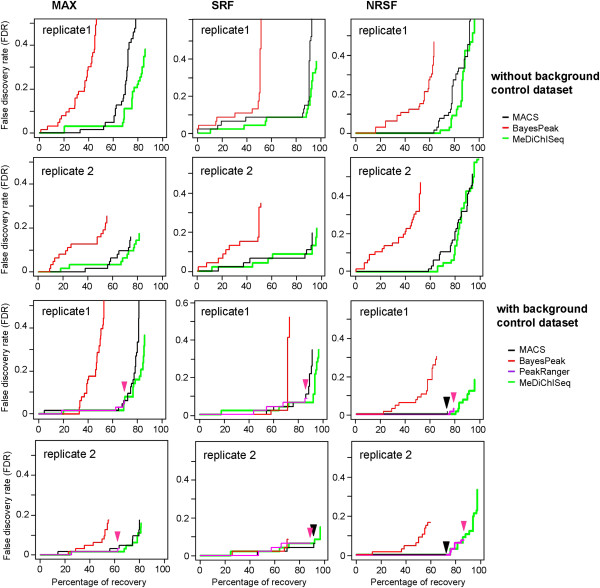
**Specificity and sensitivity of MeDiChISeq peak predictions compared with other algorithms.** A manually curated ChIP-seq benchmark dataset for MAX, SRF and NRSF [17] was used to assess the percentage of true site recovery by the indicated peak calling algorithms relative to the false discovery rate (FDR). For the two upper panels no background control sample was used during peak calling. The two lower panels show the same analysis but with considering the corresponding background control dataset in the analysis. Note that PeakRanger performance is not illustrated in the upper panels, as this algorithm does not perform IP binding site assessment without background control. In cases where the tracings overlap, an arrow indicates the point of maximal recovery.

Previous studies have evaluated peak caller performance to distinguish false positives from “true” binding sites by using a manually curated collection of binding regions (and “false” enrichment sites) that cover typical variation in peak size and appearance for the TFs NRSF, SRF and MAX [14, 17]. We have evaluated MeDiChISeq in the context of this benchmark, demonstrating in all three cases a high percentage of true binding site recovery (> 80%) and low false discovery rate (Figure 3). It is worth mentioning that, while its overall FDR performance is similar to that of MACS, MeDiChISeq generally retrieves more true binding sites. Furthermore, while using a background control dataset affected the false discovery rate of all other evaluated peak callers, MeDiChISeq performed equally well in identifying true binding events in presence and absence of this control. This is most likely due to the fact that the binding site identification relies on a pre-computed kernel and is thus less affected by artifactual enrichment events. This performance is well illustrated in the case of NRSF datasets, where in the absence of background control dataset, MACS and MeDiChISeq present a maximal percent of recovery of 90% but accompanied by high FDR levels (>0.5). Importantly, the incorporation of background control dataset in the analysis reduces the FDR levels but the percentage of true site recovery is also compromised for MACS (less than 80%), while MeDiChISeq manages to keep the percentage of recovery levels up to 90% even under these conditions (FDR < 0.4).

### Peak caller performance relative to sequencing depth

The rapid technological progress in the field of massive parallel sequencing provided over the years sequencing platforms with continuously increasing sequencing depths. In fact, while the first versions of the Illumina Genome analyzer had sequencing capacities in the range of several millions reads, the latest Hiseq2000/2500 versions provides more than 3 billion reads per flow cell. Following this continuous progress, the number of sequenced reads used per ChIP-seq assay has increased considerably. In fact, while early ChIP-seq assays generated <4 million total mapped reads (TMRs), current datasets comprise >20 million TMRs. Importantly, increasing the sequencing depth increased also the number of discovered binding sites [5, 22, 23] but only a few studies evaluated the peak caller performance for conditions with varying sequencing depths. Obviously, increasing the sequencing depth will increase both the signal and the noise levels, which could potentially affect peak caller performance.

**Figure 4:**
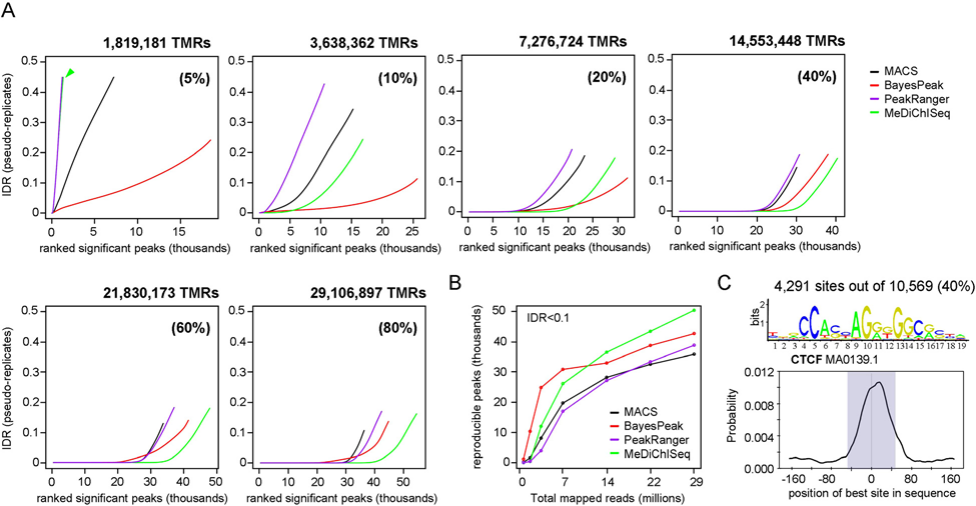
**Performance of peak calling algorithms at different sequencing depth. (A)** Pseudo-replicates with different total mapped reads (TMRs) were created from a CTCF dataset of 36,383,621 reads. Mapped reads were twice randomly sampled to obtain fractions of 5 to 80% of the original dataset, as indicated in the panels. IDR assays comparing the pseudo-replicate datasets were performed for the predictions of the four peak-calling algorithms. **(B)** The number of reproducible peaks identified for an IDR threshold of 10% (IDR < 0.1) is illustrated relative to the sequencing depth. **(C)** Motif analysis performed with the reproducible binding sites specific to MeDiChISeq (when compared with MACS) corresponding to 29,106,897 TMRs. As illustrated, more than 40% of these sites harbor a CTCF motif (top panel; Jaspar database comparison performed by CentriMO; p-value 4.4×10^–1085^) in the center of the predicted peaks (bottom panel).

To address this question, we created a high density ChIP-seq dataset by combining the datasets of the two biological CTCF replicates. This *meta*-profile comprised >36 M TMRs and was used for profile reconstruction from subsets generated by random sampling (80%; 60%; 40%; 20%; 10%; 5%) (Additional file 9A). To perform IDR evaluation, *pseudo* replicates were produced by two independent random samplings. As expected, the CTCF profile reconstructed from <2 M TMRs had unacceptably high IDR levels (Figure 4). In this condition MeDiChISeq and PeakRanger performed worst, followed by MACS and BayesPeak. This is readily explained by the fact that both MeDiChISeq and PeakRanger evaluate peak shapes, which are highly variable at low TMR levels (see *pseudo* replicates at 1.8 M TMRs in Additional file 9A). Importantly, with the increase in the TMR levels peak shapes consolidate and the performance of MeDiChISeq is enhanced accordingly (Figure 4A). Indeed, above 14 M TMRs MeDiChISeq outperforms all other peak callers with respect to the number of significant peaks with the lowest IDR levels. Note that above this TMR level all peak callers tend to reach saturation, a phenomenon generally referred to as the sequencing depth beyond which the number of newly discovered sites (in this case in a reproducible manner) reach a constant level (i.e. between 30 to 40 thousand sites for IDR levels lower than 0.1; Figure 4B).

This comparative study clearly demonstrates a direct correlation between TMR size and the degree of reproducible peak identification by any of the compared peak callers. In addition, it shows that MeDiChISeq, and to a certain degree also BayesPeak, tend to retrieve more reproducible binding sites than MACS and PeakRanger. This could be the direct consequence of different sensitivities and/or specificities of the peak callers under these conditions. To assess this issue we evaluated the extent of overlap between the retrieved sites by the different methods relative to MeDiChISeq. This analysis demonstrated that MeDiChISeq retrieved >88% of the binding sites identified by the other methods, but predicted an additional >10,000 specific sites (Additional file 9B).

These additional sites may originate from the use of the stringent comparative conditions (summit overlap +/−50nts distance). Indeed, a comparison between MACS and MeDiChISeq revealed that > 4,000 of the 15,000 MeDiChISeq-specific sites overlapped with MACS calls, when peak comparison settings were relaxed (Additional file 10). The remaining 10,000 binding sites that did not appear in the MACS-predicted site list were further evaluated for the presence of the previously described CTCF motif. Importantly, more than 4,000 MeDiChISeq-specific sites (40%) contained a CTCF motif, strongly suggesting that this population corresponds to *bona fide* CTCF binding sites that were ignored by MACS (Figure 4C). Of note, PeakRanger and/or BayesPeak identified nearly 3,000 of these *bona fide* CTCF binding sites (illustrated in Additional file 10C, right panel).

## Conclusions

Here we present MeDiChISeq, a model-based deconvolution approach to assess binding events and chromatin marks from ChIP-seq datasets. We have previously used an early version of this methodology for mapping the chromatin localization of RXRα and RARγ nuclear receptors [24], as well as for profiling RNA polymerase II [16]. This report describes the implementation of MeDiChI - originally developed by David Reiss to evaluate ChIP-chip profiles [15] – for the analysis of datasets generated by massive parallel sequencing.

From the conceptual point of view, this methodology applies a different rationale to define an enrichment event. In contrast to other peak detection algorithms, MeDiChISeq uses the binding pattern properties, inherent to the ChIP-seq profile under study, to define enrichment and background characteristics. Albeit other shape-based methodologies for binding site identification exist (e.g. Triform [14]; T-PIC [13]), MeDiChISeq presents further conceptual advantages originating from the training step that defines a “consensus” binding pattern, which is then used to identify significant binding events at genome-wide level. While a direct comparison of the various shape-based methodologies would be of interest, these tools were not operative/available when we performed this study.

The comparative analysis of MACS, BayesPeak and PeakRanger performance revealed that MeDiChISeq identifies most of the sites predicted by other methods, but in addition it discovers new significant binding events/marks with a low level of false calls. We thus conclude that the incorporation of a more complex feature to define the relevance of an enrichment event, i.e. the evaluation of its shape defined by a preliminary training process, is a major advantage for the peak calling process. While MeDiChISeq has shown also optimal performance when identifying binding patterns in histone modification marks like H3K4me3 or H3K27ac, which present broader enrichment patterns than transcription factors, we did not perform exhaustive analyses on even broader pattern profiles like those observed for H3K36me3 or H3K27me3, because the current MeDiChISeq release does not include enrichment island identification, as is the case for other tools like SICER [25], RSEG [26] or BroadPeak [27]. Nevertheless, the present release of MeDiChISeq is already able to perform optimal binding site identification also for rather broad enriched patterns, such as the H3K36me3 histone mark (Additional file 11). Importantly, such multiple site identification recapitulates the enrichment island patterns identified by SICER, strongly suggesting that also MeDiChISeq performs well in such situations. In this context, a further optional computational module that merges closely annotated binding/modification sites is being developed to use MeDiChISeq outputs for enrichment island prediction.

## Authors’ information

These authors should be regarded as joint First Authors: Marco-Antonio Mendoza-Parra and Malgorzata Nowicka.

## Electronic supplementary material

Additional file 1: **The model of a single binding event in ChIP-seq datasets.** (PDF 209 KB)

Additional file 2: **A training approach for learning optimal read elongation parameters.** (PDF 160 KB)

Additional file 3: **Characterising ChIP-seq Binding Patterns by Model-Based Peak Shape Deconvolution.** (PDF 140 KB)

Additional file 4: **MeDiChISeq vignette.** (PDF 431 KB)

Additional file 5: **MeDiChISeq manual.** (PDF 107 KB)

Additional file 6: **MeDiChISeq R package.** (ZIP 2 MB)

Additional file 7: **Peak caller characteristics and their relevant parameters used in this study.** (PDF 7 KB)

Additional file 8: **Overlapping peaks identified by different peak calling algorithms.** (PDF 16 KB)

Additional file 9: **MeDiChISeq binding sites identification at different sequencing depth levels compared with that performed by other peak caller approaches.** (PDF 187 KB)

Additional file 10: **Detailed analysis of the binding sites identification performed by MeDiChISeq and MACS in a CTCF ChIP-seq profile reconstructed from 29’100,000 TMRs.** (PDF 109 KB)

Additional file 11: **H3K36me3 profiling analyzed by MeDiChISeq and compared with the cluster identification approach SICER.** (PDF 24 KB)
