# Supplementary material for: Characterising ChIP-seq binding patterns by model-based peak shape deconvolution
Source: BMC Genomics. 2013 Nov 26;14(1):834. doi: 10.1186/1471-2164-14-834 (PMC4046686; doi:10.1186/1471-2164-14-834)
Supplement: Supplementary file 1 — Additional file 1: The model of a single binding event in ChIP-seq datasets. (PDF 209 KB) [file 12864_2013_5524_MOESM1_ESM.pdf]

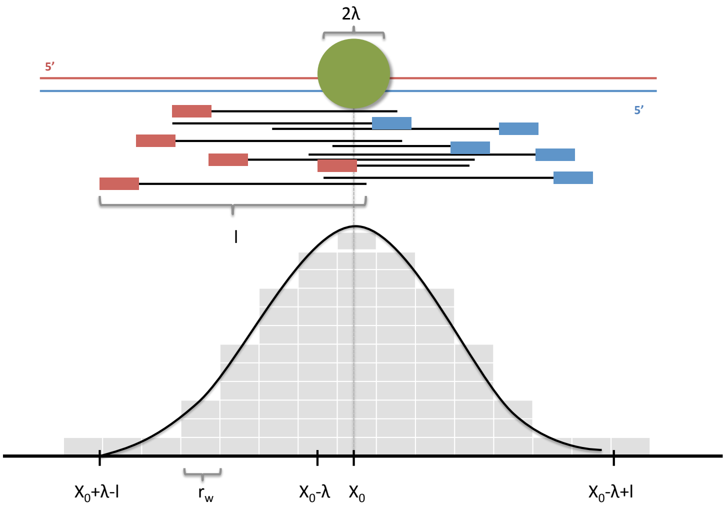

Additional file 1. The model of a single binding event in ChIP-seq data.
Here we present the ChIP-seq adjustment of the model for a single protein DNA binding event that was originally created for ChIP-chip assays [1]. Because of the difference in technology used to assess the abundance of specific bindings, the interpretation of some of the model parameters changes. For instance in ChIP-seq assays there is no hybridization of DNA fragments to the probes. Nevertheless peak shape patterns retrieved in ChIP-seq datasets can be modeled in a similar manner as done for ChIP-chip datasets, see Fig. 1 in [1]. Let's introduce the following parameters:

- the wig resolution r_w,
- the footprint 2λ of the DNA binding protein on the DNA,
- the distribution $f(l)$ of lengths of DNA fragments that were bounded by the protein of interest,
- the $\Phi(\nu)$ is a helper function for counting how many fragments overlap with given WIG window, $\Phi(\nu) = \mathbb{1}(\nu > 0)$.

Note: Previously defined function $I(l)$ of fragment intensities does not appear in sequencing model since there is no fluorescent intensities measured, so we make it equal 1.
A DNA fragment of length l that was generated from a region where the binding took place at the genomic coordinate x_0 can begin at any coordinate within the interval $[x_0 + \lambda - l; x_0 - \lambda]$. Such fragment can overlap with any genomic position within $[x_0 + \lambda - l; x_0 - \lambda + l]$ and it will be counted in each WIG window which center lies in the range $[x_0 + \lambda - l - r_w/2; x_0 - \lambda + l + r_w/2]$. The probability that a fragment will be counted in the WIG window centered at position x is:

$$P_l(x - x_0) = \frac{1}{l - 2\lambda} \sum_{i=\lambda-l}^{-\lambda} \Phi\left(\sum_{j=i}^{l+i} H(j, x - x_0)\right),$$

$$H(j, x - x_0) = \begin{cases} 1 & \text{if } j \in [x - x_0 - r_w/2; x - x_0 + r_w/2] \\ 0 & \text{othervise} \end{cases}$$

The final intensity profile is of the form:

$$K(x - x_0) = \sum_l f(l) P_l(x - x_0).$$

As it is described in details in [1] the shape of kernel K is mostly influenced by the fragment length distribution $f(l)$ which is parametrized with Gamma distribution $\Gamma(\alpha, \beta)$. Thus only α and β parameters are necessary to define kernel function for a given data set.

References

[1] David J. Reiss, Marc T. Facciotti, and Nitin S. Baliga. Model-based deconvolution of genome-wide dna binding. *Bioinformatics*, 2007. doi: 10.1093/bioinformatics/btm592.
