# Supplementary material for: Characterising ChIP-seq binding patterns by model-based peak shape deconvolution
Source: BMC Genomics. 2013 Nov 26;14(1):834. doi: 10.1186/1471-2164-14-834 (PMC4046686; doi:10.1186/1471-2164-14-834)
Supplement: Supplementary file 2 — Additional file 2: A training approach for learning optimal read elongation parameters. (PDF 160 KB) [file 12864_2013_5524_MOESM2_ESM.pdf]

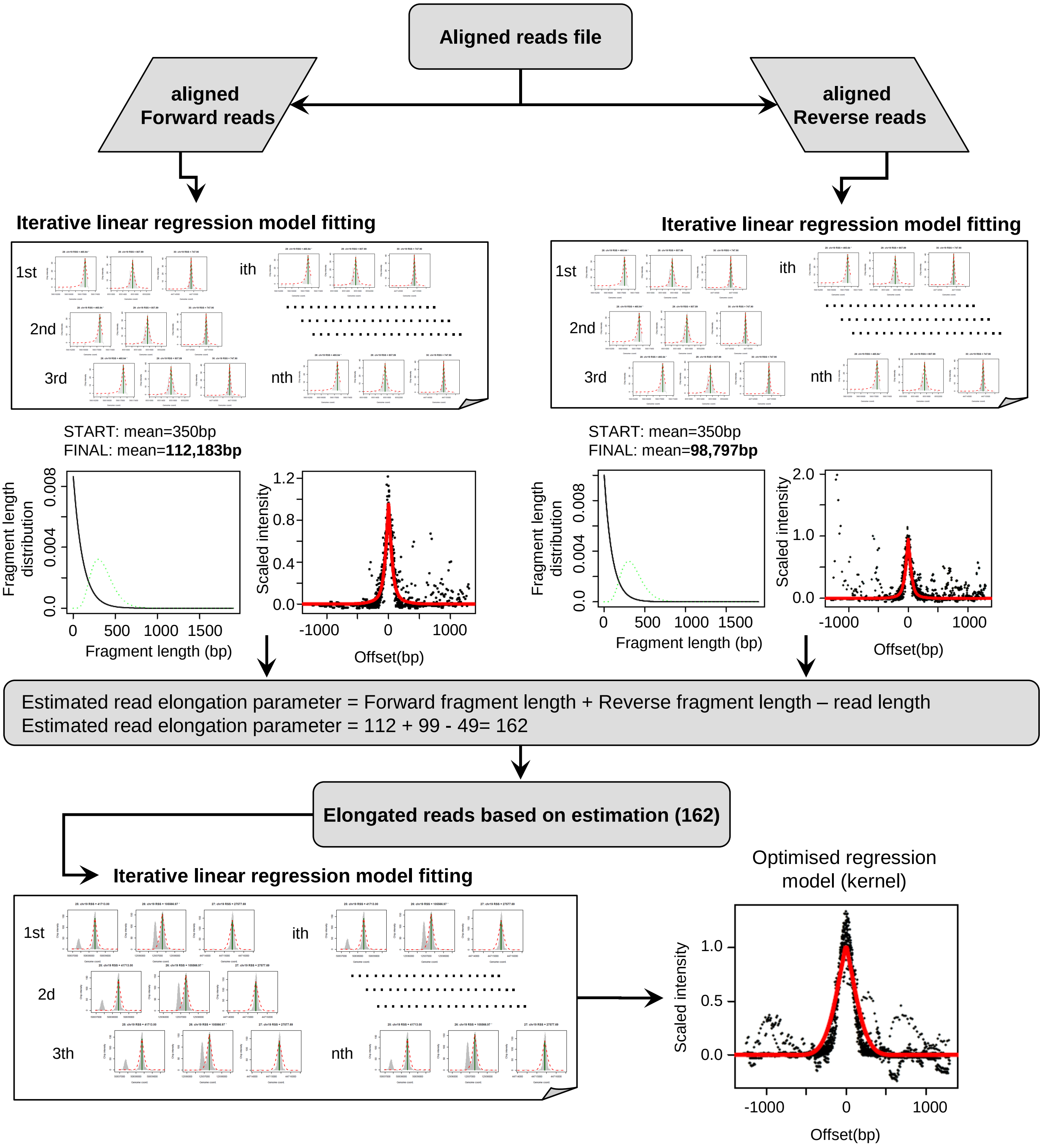

Additional file 2. Schematic representation illustrating the approach implemented in MeDiChISeq for estimating the read elongation parameter. In cases where the user is not able to propose a read elongation parameter from DNA fragmentation information, MeDiChISeq uses iterative linear regression model fitting in a strand-specific mode. From this preliminary step, the fragment length per strand is inferred, then the estimated read elongation parameter is estimated by adding the forward and reverse fragment length and subtracting the read length. In the illustrated example (CTCF dataset), this analysis estimated a read elongation parameter of 162bp.
