# Supplementary material for: Characterising ChIP-seq binding patterns by model-based peak shape deconvolution
Source: BMC Genomics. 2013 Nov 26;14(1):834. doi: 10.1186/1471-2164-14-834 (PMC4046686; doi:10.1186/1471-2164-14-834)
Supplement: Supplementary file 3 — Additional file 3: Characterising ChIP-seq Binding Patterns by Model-Based Peak Shape Deconvolution. (PDF 140 KB) [file 12864_2013_5524_MOESM3_ESM.pdf]

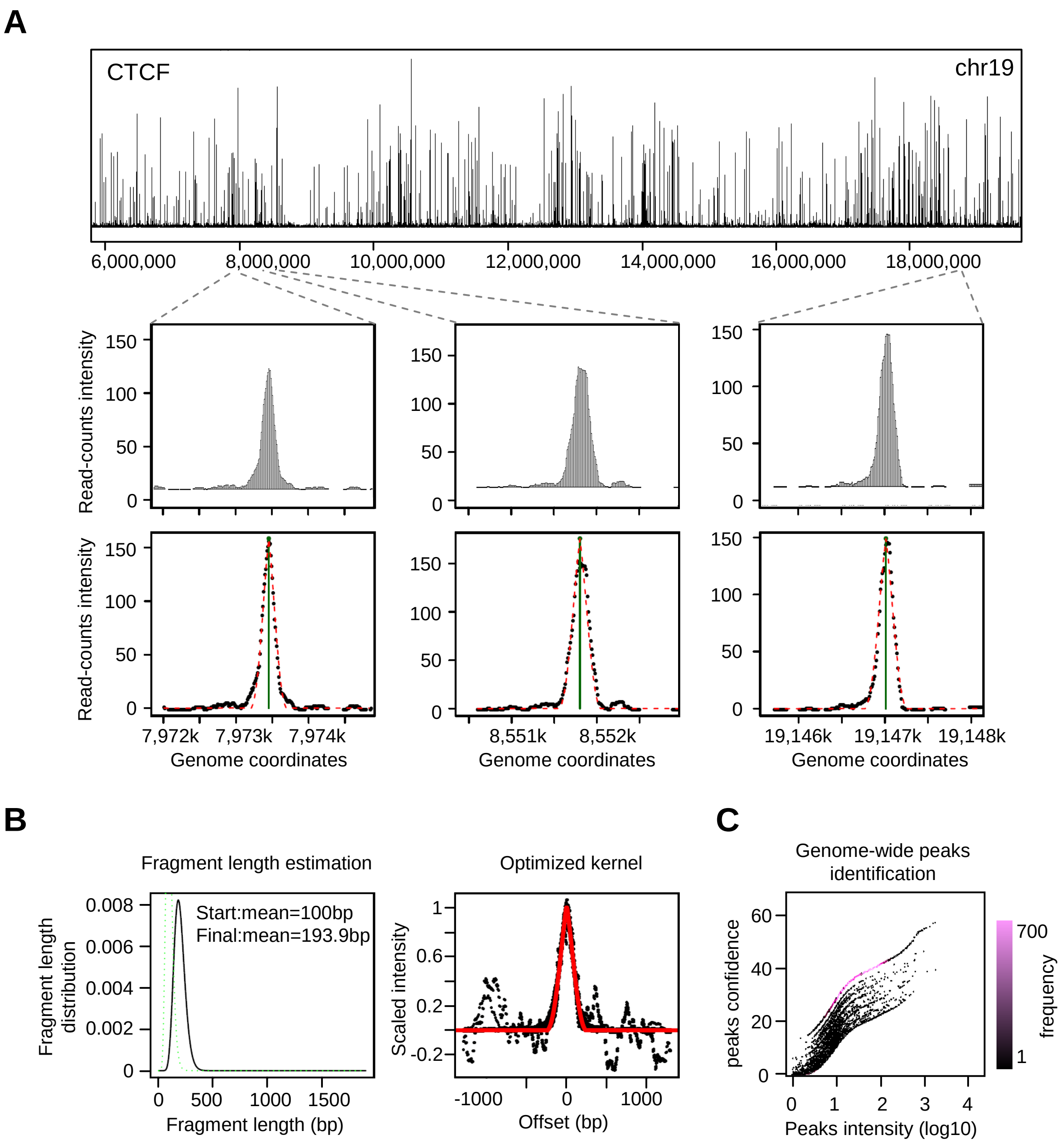

Additional file 3. Characterising ChIP-seq binding patterns by model-based peak shape deconvolution. (A) MeDiChISeq apply first a training process on selected high read-count intensity regions. This process consist in applying a linear regression model fitting in an iterative manner. (B) After a certain number of iterations, the training process defines the best regression model (kernel) representative of the evaluated binding patterns. This model is also used to infer the DNA fragment length from the base of the enrichment patterns defined by the optimized kernel (C) The optimized kernel is applied to identify genome-wide binding/modification patterns.
