# Supplementary material for: Characterising ChIP-seq binding patterns by model-based peak shape deconvolution
Source: BMC Genomics. 2013 Nov 26;14(1):834. doi: 10.1186/1471-2164-14-834 (PMC4046686; doi:10.1186/1471-2164-14-834)
Supplement: Supplementary file 4 — Additional file 4: MeDiChISeq vignette. (PDF 431 KB) [file 12864_2013_5524_MOESM4_ESM.pdf]

The MeDiChISeq ChIP-seq deconvolution package

Malgorzata Nowicka and Wouter Van Gool
under the direction of
Marco Antonio Mendoza Parra and Hinrich Gronemeyer

August 12, 2013

Contents

1 Technical notes **1**
1.1 Installation . . . 1
1.2 Input data . . . 2
1.3 Parallelization . . . 2
1.4 Clonal reads . . . 2

2 Learning a representative binding pattern **2**

3 Whole genome deconvolution **5**

4 Single window deconvolution **8**

5 Choosing an appropriate threshold **10**

1 Technical notes

MeDiChISeq package is an adaptation of previously described MeDiChI, Reiss et al. (2007), a deconvolution package for ChIP-chip assays. MeDiChISeq has been previously used in Mendoza-Parra et al. (2011) and Mendoza-Parra et al. (2012) in the context of ChIP-seq datasets.

1.1 Installation

Part of MeDiChISeq functions is written in C++ and integrated with R via Rcpp package. This requires the installation of boost library, which on Linux system can be done with the command `sudo apt-get install libboost-all-dev`. Additionally parallel

package for parallel computation and packages lars, quadprog and corpcor are required. MeDiChISeq can be loaded as follows:

```
> library(MeDiChISeq)
```

1.2 Input data

The example data set used in this tutorial has been originally published by Ernst et al. (2011) and can be downloaded from the public repository GEO (GSM646314, GSM646332, GSM646424 and GSM646430) and from MeDiChiSeq website together with the peak calling results. We illustrate how to annotate peaks in ChIP-seq profiles such as CTCF, which presents sharp binding patterns, and H3K4me3 that is characterized by broader binding islands.

While in the examples illustrated in this vignette we have used BED format files as input datasets, MeDiChISeq can process mapped read files in following formats: SAM, BAM, BOWTIE, SOAP.

1.3 Parallelization

Due to the important computation requirements during the linear regression fitting and the bootstrapping large datasets might require several hours of processing. For this reason both `fit.peak.profile.seq` and `deconv.entire.genome.seq` functions can be run following a multicore mode. The parallelization schema is based on `mclapply` function from parallel package, thus users can specify the number of processors in use with the `nr.cores` parameter (see below).

1.4 Clonal reads

It can happen that the ChIP-seq datasets may contain an important fraction of sequenced reads aligned to the exactly same genomic position. Such so called clonal reads are usually considered as artefacts resulting from the PCR amplification step retrieved in most of the library construction protocols. If this is a frequent phenomena (i.e. the fraction of clonal reads is significant relative to the total population) they can have a negative influence on the shape of peaks. That is why we suggest to remove them using the parameter `remove.clonal.reads=TRUE`, which abrogates the clonal population, but keeps a copy of them defined by the parameter `clonal.reads.to.keep`.

2 Learning a representative binding pattern

In the first step MeDiChISeq defines a representative binding pattern from the provided ChIP-Seq dataset. In fact, this step is justified by the fact that several technical aspects in the generation of a given ChIP-seq dataset, among them the chromatin sonication,

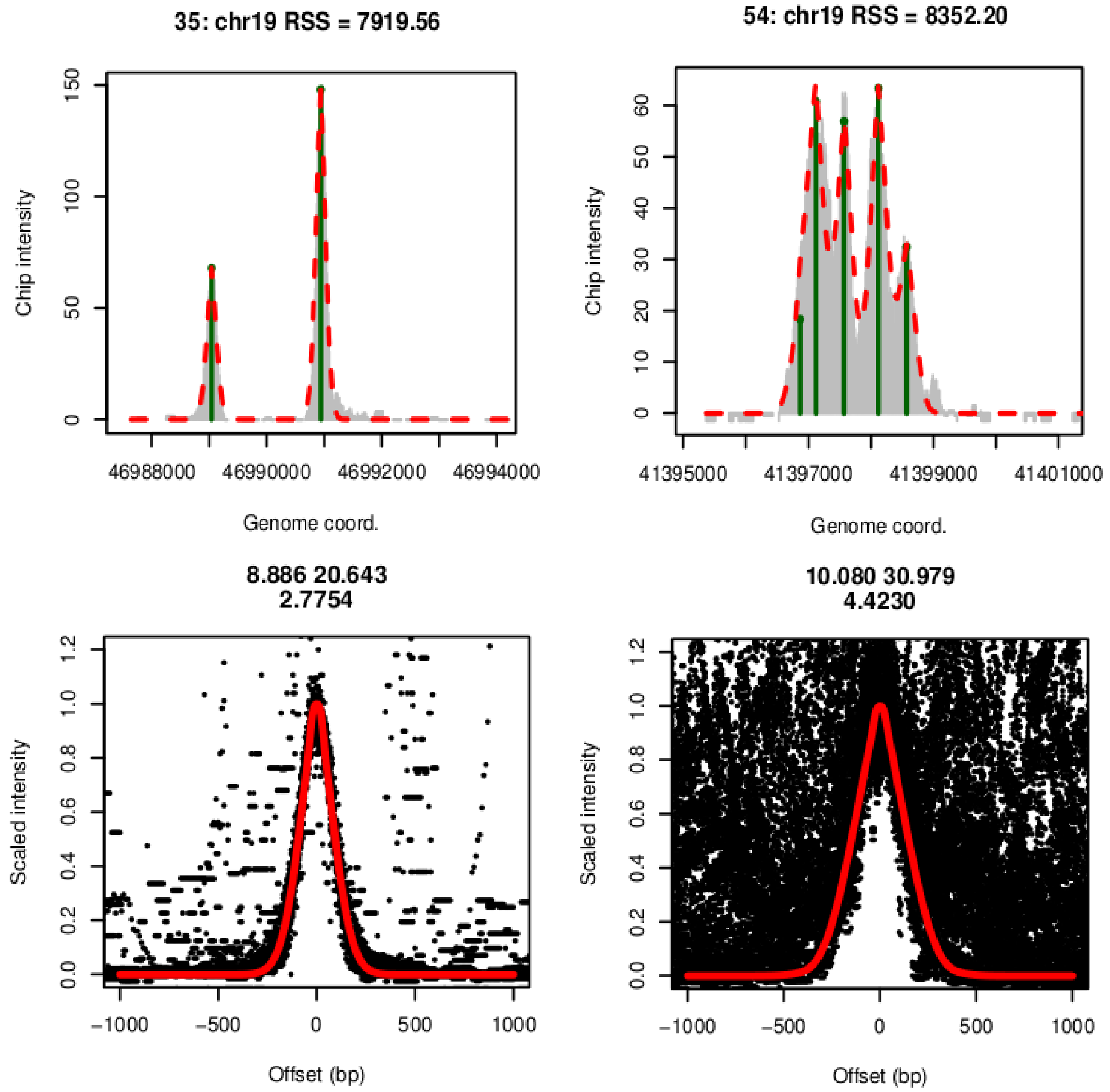

Figure 1: Example of highest intensity peaks used to learn the kernel shape and the final fitting for CTCF (left) and H3K4me3 (right) profiles.

but also the inherent nature of the factor under study (i.e. sharp versus broad binding pattern profiles) may directly influence the shape of peaks populating the ChIP-seq profile.

Briefly, this is performed by fitting a binding pattern model, described here as kernel, to a reduced number of genomic regions, for instance those retrieved in a single chromosome. The formalism of this procedure is extensively described in Reiss et al. (2007). Such kernel is used in a second step of MeDiChISeq work flow for identifying binding

events genome wide.

The shape of binding sites is learned with `fit.peak.profile.seq` function. Starting with a default kernel, peaks are iteratively annotated and refitted and kernel parameters are adjusted in order to find the modeled shape that will fit the best to the observed one. We assume that in general high intensity peaks represent true binding sites, which is why refitting and learning of new parameters takes place only in such regions. The number of regions to consider can be defined with `n.peaks` and `n.skip` parameters. Note that the threshold (`quant.cutoff = "q1e-7"`) for annotating putative binding regions can be more stringent than in `deconv.entire.genome.seq` (`quant.cutoff = "q1e-5"`) because we are interested in annotating a fraction of binding sites for learning the representative binding pattern.

Currently, parameters that are optimized include the shape and scale of the Gamma function used to asses the fragment length distribution. The choice of starting parameters can influence the final fitting, thus it is important to choose the one that could be close to the expected final results. After performing multiple fittings on different profiles we suggest to use `start.pars = c(shape = 10, scale = 30)` which correspond to the average DNA fragment length of 300 bp.

In the example below short reads are first elongated by `reads.elong=150` bp, then the kernel fitting is performed. This elongation parameter should correspond more or less to the sonication fragment length. If we have no feeling about value to set up, we can define `reads.elong=NULL`, then the elongation distance will be estimated from forward and reverse read profiles. This is performed as following: kernel is fitted separately to the forward and reversed intensity profiles, which are created from reads of length `reads.length`. Then `reads.elong` equals to

$$estimated.fragment.length_{Forward} + estimated.fragment.length_{Reverse} - reads.length.$$

Nevetheless we suggest to use the first approach since it is faster and gives optimal results for most of the evaluated profiles.

This is an example of code that will find the kernel fitting to the CTCF profile, figure 1.

```
> file.IP <- "GSM646314_GM12878_CTCF_rep1.bed"
> fit.peak.profile.seq(file.IP, format="bed", genome="hg18", output.dir=NULL,
+ output.name="CTCF", chrom.fit="chr19", limL=0, limU=Inf,
+ reads.elong=150, quant.cutoff="q1e-7", window=50000,
+ mini.window=3000, wig.res=10, fit.res=50, reads.length=50,
+ n.peaks = 50, n.skip = 20, re.fit=100, max.iter=500, selection.method="bic",
+ post.proc.factor=2, start.pars = c(shape =10, scale = 30),
+ to.be.fit=c("shape", "scale"), method = "L-BFGS-B",
+ nr.cores=1, remove.clonal.reads=TRUE, clonal.reads.to.keep=3,
+ write.pdf=TRUE, save.kernel=TRUE, verbose.console=TRUE,
+ overwrite.wigs=FALSE,  keep.wigs=TRUE)
```

As most of the parameters can be set up to their default values, the same results can be achieved with much simpler code:

```
> fit.peak.profile.seq(file.IP, genome="hg18",
+ output.name="CTCF", chrom.fit="chr19",
+ quant.cutoff="q1e-7", window=50000, mini.window=3000,
+ start.pars = c(shape =10, scale = 30),
+ method = "L-BFGS-B")
```

With the same settings we can find the kernel for broad H3K4me3 profile, figure 1.

```
> file.IP <- "GSM646424_Huvec_H3K4me3_rep1.bed"
> fit.peak.profile.seq(file.IP, genome="hg18",
+ output.name="H3K4me3", chrom.fit="chr19",
+ quant.cutoff="q1e-7", window=50000, mini.window=3000,
+ start.pars = c(shape =10, scale = 30),
+ method = "L-BFGS-B")
```

The output of `fit.peak.profile.seq` consists of the list of following objects:

- `reads.elong`: short reads were elongated to this length in order to create the intensity WIG files on which the fitting is performed.
- `kernel`: final peak profile.
- `frag.length`: estimated fragment length. From the kernel model, the base of peaks corresponds to 2∗`frag.length`.

Moreover, if `kernel=TRUE, verbose.console=TRUE, keep.wigs=TRUE`, than in the directory `output.dir` all the above outputs will be saved together with generated WIG file, console display of work progress and the PDF files with progress of kernel fitting and final kernel plots.

3 Whole genome deconvolution

In this part, we will deconvolve a small chunk of chromosome 19 from CTCF data set. In general we can specify any genome piece of interest with parameters `chrom.list, limL, limU, potential.peaks`. Such region is divided into overlapping windows which are further deconvolved with adjusted version of `chip.deconv` function from MeDiChI package. For the whole genome deconvolution set `chrom.list=NULL, limL=0, limU=Inf, potential.peaks=NULL`, which is the default.

When required the `potential.peaks` parameter makes reference to an output in BED format from any other peak caller, for instance MACS, whose results would be validated using MeDiChISeq. Alternatively, it could be used to target MeDiChISeq analysis

to different chromatin regions, for instance promoter loci, by providing their coordinates in BED format.

Remember to set up `reads.elong` to the value that had been used for elongation when the chosen kernel was produced by `fit.peak.profile.seq` function.

When `nr.boots` ≥ 2, for each peak global and local p-values are calculated. Using `local.windows` parameter define the number and the size of areas surrounding each peak that we consider in calculations of local confidence. Final p-values are a combination of all local and global p-values.

In this example we also introduce the control sample which is deconvolved in parallel with IP and used for correcting the confidence of peaks in case when the control sample presents enriched patterns in the same chromatin loci. Parameter `Control.corr.param` defines the minimal percentage of overlap between peaks from IP and control to consider them in the same loci and apply correction.

When `verbose.console=TRUE` we can follow progress of deconvolution.

```
> file.IP <-  "GSM646314_GM12878_CTCF_rep1.bed"
> file.Control <-  "GSM646332_GM12878_WCE_rep1.bed"
> reads.elong <- "MeDiChISeq_CTCF_reads.elong.txt"
> kernel <- "MeDiChISeq_CTCF_kernel.txt"
> frag.length <- "MeDiChISeq_CTCF_frag_length.txt"
> deconv.entire.genome.seq(file.IP, file.Control=file.Control, format="bed",
+ genome="hg18", output.dir=NULL, output.name="CTCF",
+ chrom.list=NULL, limL=0, limU=Inf, potential.peaks=NULL,
+ reads.elong=150, kernel=kernel, frag.length=frag.length,
+ quant.cutoff="q1e-5", window=20000, wig.res=10,
+ fit.res=50, max.steps=100, selection.method="bic",
+ post.proc.factor=2, nr.boots=5,
+ local.windows=c(1000, 2000, 5000), Control.corr.param=0.01,
+ nr.cores=1, remove.clonal.reads=F, clonal.reads.to.keep=3,
+ verbose.console=TRUE, overwrite.wigs=FALSE, keep.wigs=TRUE)
```

Most of the parameters are set up to their default values and above run is equivalent to the followig one:

```
> deconv.entire.genome.seq(file.IP, file.Control=file.Control,
+ genome="hg18", output.name="CTCF",
+ window=20000,
+ reads.elong=reads.elong, kernel=kernel,
+ frag.length=frag.length,
+ nr.cores=1, remove.clonal.reads=F)
```

For profiles with broad patterns we suggest to use a bigger `window=50000` in order to properly estimate the background which can not be ”caught” when the window is too small.

```
> file.IP <-  "GSM646424_Huvec_H3K4me3_rep1.bed"
> file.Control <-  "GSM646430_Huvec_WCE_rep1.bed"
> reads.elong <- "MeDiChISeq_H3K4me3_reads.elong.txt"
> kernel <- "MeDiChISeq_H3K4me3_kernel.txt"
> frag.length <- "MeDiChISeq_H3K4me3_frag_length.txt"
> deconv.entire.genome.seq(file.IP, file.Control=file.Control,
+ genome="hg18", output.name="H3K4me3",
+ window=50000,
+ reads.elong=reads.elong, kernel=kernel,
+ frag.length=frag.length,
+ nr.cores=1, remove.clonal.reads=F)
```

Generated output consists of the following object:

- `All.coeffs.IP` that contains a list of all annotated peaks with their intensities, global, local and combined p-values and coefficients after control correction.

```
> out.CTCF <- read.table("MeDiChISeq_CTCF_ALL_COEFFS_IP.txt", head=T)

> head(out.CTCF)

  chromosome  start    end position intensity local.p.value.1000
1       chr1    218    584      401  16.97723                0.2
2       chr1    968   1334     1151 330.18133                0.2
3       chr1  15768  16134    15951  35.36577                0.2
4       chr1  81068  81434    81251  78.59003                0.2
5       chr1  94668  95034    94851  17.27626                0.2
6       chr1 128678 129044   128861  12.45522                0.2
  local.p.value.2000 local.p.value.5000 global.p.value
1                0.8                0.8   0.0083947686
2                0.2                0.2   0.0001646784
3                0.2                0.2   0.0027267679
4                0.2                0.2   0.0010512609
5                0.2                0.2   0.0080998794
6                0.2                0.2   0.0170557034
  combined.local.global.p.values if.control.overlap control.correction
1                   8.419882e-03                  1          -5.177828
2                   1.915517e-05                  1        1554.725293
3                   2.559443e-04                  0         127.028713
4                   1.066910e-04                  0         312.149553
5                   6.897352e-04                  0          54.615740
6                   1.350755e-03                  0          35.739306
```

This object is saved in `output.dir`. Moreover you can find there a BED file with annotated peaks and their intensities and WIG files which can be loaded to any genome browser for visual verification of obtained results. In order to facilitate the choice of p-values cutoff a plot of intensities versus p-values is generated, for more details go to section 5. The console output of `deconv.entire.genome.seq` is also saved.

4 Single window deconvolution

Here we present the use of the core function `chip.deconv.seq` which deconvolves the subset of ChIP-seq intensity profile and allows to get a visual representation of deconvolution in a given window, see figure 2. This function and its adjusted version are the slight modifications of `chip.deconv` designed by (Reiss et al., 2007).

`chip.deconv.seq` works over any WIG intensity file that can be generated with `write.wigs.parallel` function.

Deconvolution of one (centered in 461582) of the windows produced by `deconv.entire.genome.seq` for CTCF - sharp pattern profile.

```
> data <- read.table("chr19_IP_GSM646314_GM12878_CTCF_rep1_res-10_dist-150_both.wig",
+                     skip=2)
> kernel <- read.table("MeDiChISeq_CTCF_kernel.txt")

> out <- chip.deconv.seq(data = data, center = 461582, window = 20000,
+ kernel = kernel, quant.cutoff = "q1e-5", fit.res = 50)

Using 17 as data cutoff!
Step for min AIC: 19 13 ; BIC: 19 13 ; using: bic
After LARS step: Number of coeffs: 12
After POST.PROC step: Reduced to 3 non-redundant coeffs.
After SOLVE.QP step: Reduced to 3 coeffs.

> coef(out)

   position  intensity
5    457891 128.747051
9    458091   6.271881
16   461791 177.337482

> plot(out)

> plot(out, center=457891, window=3000)
```

Deconvolution of one (centered in 469384) of the windows produced by `deconv.entire.genome.seq` for H3K4me3 - broad pattern profile.

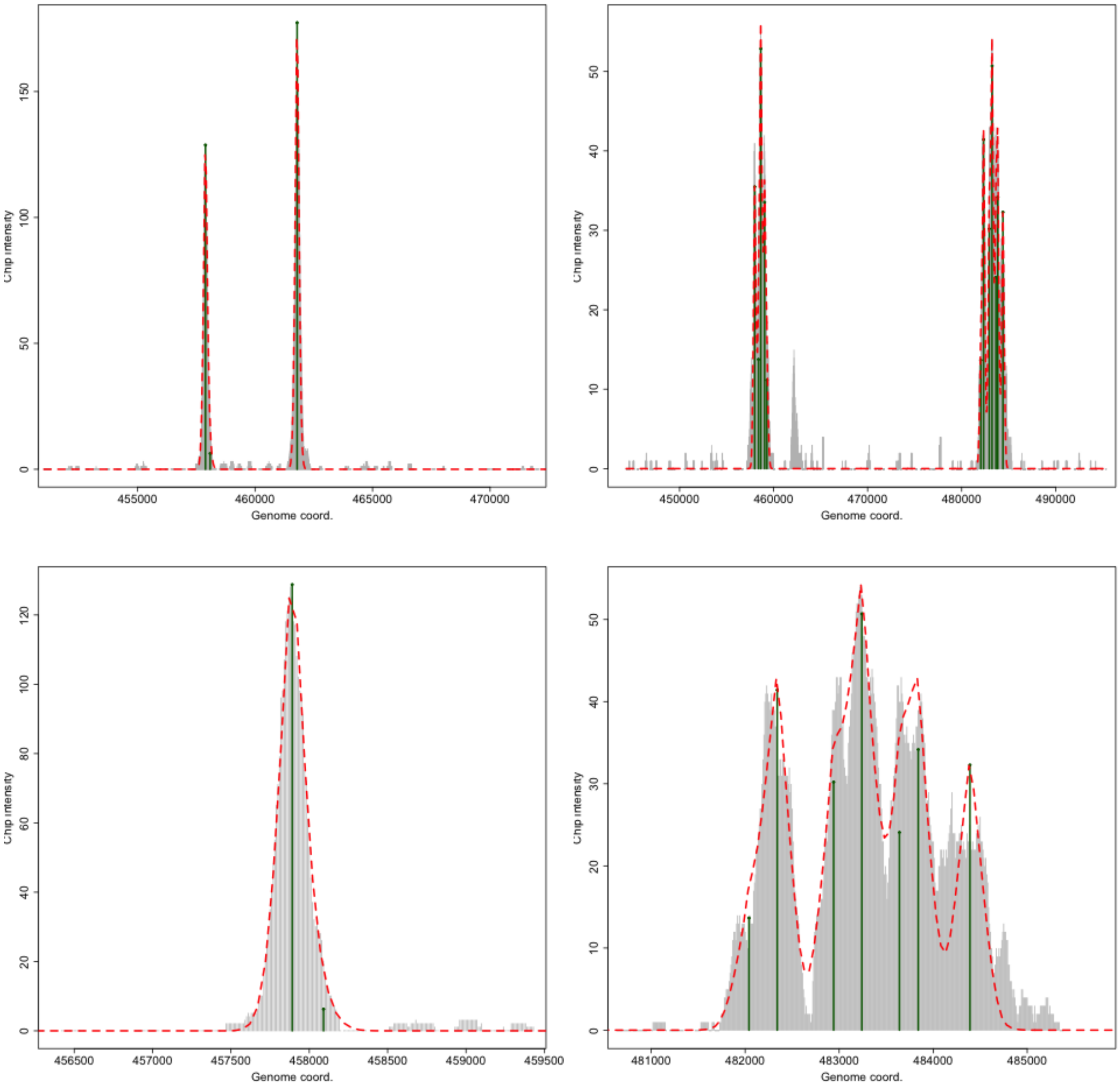

Figure 2: Single window deconvolution of CTCF (left) and H3K4me3 (right) profiles. Top panels present the results for the entire window. Bottom panels are zoomed on one of the deconvolved regiones.

```
> data <- read.table("chr19_IP_GSM646424_Huvec_H3K4me3_rep1_res-10_dist-150_both.wig"
+                     skip=2)
> kernel <- read.table("MeDiChISeq_H3K4me3_kernel.txt")
```

```
> out <- chip.deconv.seq(data = data, center = 469384, window = 50000,
+ kernel = kernel, quant.cutoff = "q1e-5", fit.res = 50)
```

```
Using 17 as data cutoff!
```

```
Step for min AIC: 84 37 ; BIC: 83 35 ; using: bic
After LARS step: Number of coeffs: 34
After POST.PROC step: Reduced to 16 non-redundant coeffs.
After SOLVE.QP step: Reduced to 16 coeffs.
```

```
> coef(out)
```

```
   position intensity
6    439741 52.013324
12   440041 12.647778
13   440491  5.323957
16   440641 24.842739
23   457991 35.477587
30   458391 13.749030
35   458641 52.863271
43   459041 33.524107
48   459291 11.134761
49   482041 13.655591
55   482341 41.424502
63   482941 30.200085
69   483241 50.693223
77   483641 24.081201
81   483841 34.167882
92   484391 32.286787
```

```
> plot(out)
```

```
> plot(out, center=483241, window=5000)
```

5 Choosing an appropriate threshold

At this stage an output of `deconv.entire.genome.seq` consists of a list of all putative peaks even those with low confidence and final elimination of those false positives should be applied. On the zoomed deconvolution window for CTCF (figure 2, lower panel) we can see that two peaks were called including one (position 458091) with very low intensity (6.271881) which is a result of overfitting and looks rather like a shoulder of the peak annotated in position 457891. As shown further this low intensity peak gets higher p-value ($6.767360e-03$) and its control correction coefficient, defined as

$$-\log 10(combined.local.global.p.values_{IP}) \times \log 10(intensity_{IP}) -$$

$$(-\log 10(combined.local.global.p.values_{Control}) \times \log 10(intensity_{Control})),$$

is much smaller (13.60735) than for the "real" peaks.

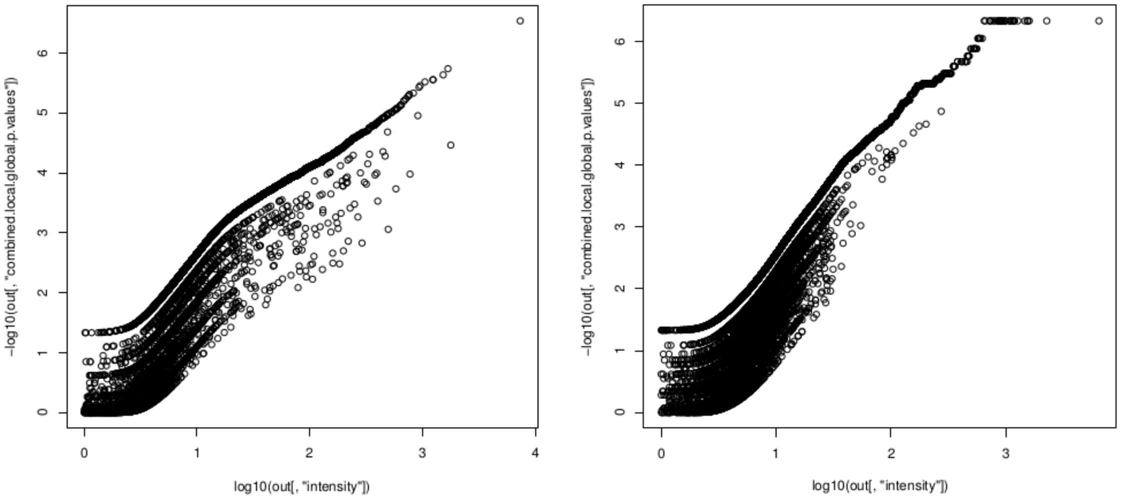

Figure 3: Logarithm of intensity versus negative logarithm of combined local and global p-values of putative peaks for CTCF (left) and H3K4me3 (right) profiles.

One can also use scatterplots of p-values versus intensity(figure 3) in order to see the distribution of those values in a given profile and choose an adequate cutoff. In the illustrated scatterplot points, which correspond to annotated binding sites, follow an S-shape distribution. This distribution is a consequence of the presence of three kinds of peaks: those with low confidence that have intensity comparable with the background; sites presenting a correlative increase between intensity and confidence and a small subset of peaks for which the confidence gets constant level though their intensity increase. Based on such scatterplots we suggest to remove the low confidence population by selecting a -log10(p-value) threshold in the range of $2 - 3$.

```
> out.CTCF[out.CTCF[,"chromosome"] == "chr19" &
+ out.CTCF[,"position"] <= 461791 & out.CTCF[,"position"] >= 457891, ]

   chromosome  start    end position  intensity local.p.value.1000
8       chr19 457708 458074   457891 128.747051                0.2
9       chr19 457908 458274   458091   6.271881                0.2
10      chr19 461608 461974   461791 177.337482                0.2
   local.p.value.2000 local.p.value.5000 global.p.value
8                 0.2                0.2   0.0006031825
9                 0.2                0.2   0.1046186546
10                0.2                0.2   0.0003925473
   combined.local.global.p.values if.control.overlap control.correction
8                    6.389683e-05                  0          540.03217
```

9	6.767360e-03	0	13.60735
10	4.293264e-05	0	774.47045

References

Jason Ernst, Pouya Kheradpour, Tarjei S. Mikkelsen, Noam Shoresh, Lucas D. Ward, Charles B. Epstein, Xiaolan Zhang, Li Wang, Robbyn Issner, Michael Coyne, Manching Ku, Timothy Durham, Manolis Kellis, and Bradley E. Bernstein. Mapping and analysis of chromatin state dynamics in nine human cell types. *Nature*, 473 (7345):43–49, May 2011. ISSN 1476-4687. doi: 10.1038/nature09906. URL `http://dx.doi.org/10.1038/nature09906`.

Marco A. Mendoza-Parra, Mannu Walia, Martial Sankar, and Hinrich Gronemeyer. Dissecting the retinoid-induced differentiation of F9 embryonal stem cells by integrative genomics. *Molecular systems biology*, 7, 2011. ISSN 1744-4292. doi: 10.1038/msb.2011.73. URL `http://dx.doi.org/10.1038/msb.2011.73`.

Marco A. Mendoza-Parra, Martial Sankar, Mannu Walia, and Hinrich Gronemeyer. POLYPHEMUS: R package for comparative analysis of RNA polymerase II ChIP-seq profiles by non-linear normalization. *Nucleic Acids Research*, 40(4):e30, February 2012. ISSN 1362-4962. doi: 10.1093/nar/gkr1205. URL `http://dx.doi.org/10.1093/nar/gkr1205`.

David J. Reiss, Marc T. Facciotti, and Nitin S. Baliga. Model-based deconvolution of genome-wide DNA binding. *Bioinformatics*, pages btm592+, 2007. doi: 10.1093/bioinformatics/btm592. URL `http://bioinformatics.oxfordjournals.org/cgi/content/abstract/btm592v1`.
