# Supplementary material for: Characterising ChIP-seq binding patterns by model-based peak shape deconvolution
Source: BMC Genomics. 2013 Nov 26;14(1):834. doi: 10.1186/1471-2164-14-834 (PMC4046686; doi:10.1186/1471-2164-14-834)
Supplement: Supplementary file 5 — Additional file 5: MeDiChISeq manual. (PDF 107 KB) [file 12864_2013_5524_MOESM5_ESM.pdf]

Package ‘MeDiChISeq’

August 12, 2013

Type Package

Title Model-based peak caller for epigenomic profiles

Version 1.0.7

Date 2013-08-09

Author Malgorzata Nowicka and Wouter Van Gool under the direction of Marco Antonio Mendoza Parra and Hinrich Gronemeyer

Maintainer Malgorzata Nowicka <gosia.nowicka@uzh.ch>

Description Self-learning, model-based peak caller

License GPL

LazyLoad yes

Depends R (>= 2.15.1), Rcpp (>= 0.10.4), parallel (>= 3.0.1), lars (>= 1.2), quadprog (>= 1.5-5), corpcor (>= 1.6.6)

Suggests lattice (>= 0.20-15), Matrix (>= 1.0-12), zoo (>= 1.7-10)

LinkingTo Rcpp

R topics documented:

chip.deconv.seq 2
deconv.entire.genome.seq 4
fit.peak.profile.seq 7
remove.clons 11
write.wigs.parallel 12

Index **14**

chip.deconv.seq *High-resolution model-based deconvolution of ChIP-seq data in WIG format.*

Description

Deconvolves a subset of data on one chromosome. Used to deconvolve an entire data set across multiple chromosomes, see `deconv.entire.genome.seq`.

Usage

```
chip.deconv.seq(data, center = NA, window = 20000, kernel = NA,
quant.cutoff = "q1e-5", fit.res = 50,
max.steps = 100, post.proc.factor = 2, selection.method = "bic",
verbose = T, trace = F, nr.boots = 1,
boot.sample.opt = "residual", max.peak = NA, boot.vary.res = F,
tile.distance = NA, where = NA,
min.npeaks = 0, max.npeaks = 99999, ...)
```

Arguments

`data` `data.frame` or `matrix` containing genome coordinates (column 1) and intensities (column 2), or a path to a WIG file.

`center` Central coordinate of the data subset to deconvolve.

`window` Size of the data chunk to deconvolve.

`kernel` `data.frame` or `matrix` which is one of the outputs of `fit.peak.profile.seq`, describing the peak shape.

`quant.cutoff` Intensity or quantile cutoff for data to be processed. Limits the locations of potential sites to those that are above this threshold.

`fit.res` Numeric for base pairs. Desired deconvolution resolution.

`max.steps` Limit the number of LARS steps taken.

`post.proc.factor` Post-processing filter for combining deconvolution coefficients. When equal 2, means that all the coefficients that are separeted by maximum 2*`fit.res` will be merged.

`selection.method` In LARS regression the accurate model is chosen based on AIC or BIC criterion. Default is `"bic"`.

`verbose` If `TRUE`, print out status messages.

`trace` If `TRUE`, print out LARS progress.

`nr.boots` Number of bootstrap iterations to perform. Attention: in contrast to `chip.deconv`, this version does not produce local p-values at all, since they are calculated in different manner by `deconv.entire.genome.seq`. See details.

`boot.sample.opt` Bootstrap resampling option. See Details. Default is `"residual"`.

`max.peak` Any coefficient with an intensity above this threshold is set to this value. Default is `NA` - no cutoff.

`boot.vary.res`	If `TRUE`, vary the resolution during bootstraps. By default `FALSE`.
`tile.distance`	Distance in bp which for ChIP-seq data corresponds to the WIGS resolution. If `NA` (the default) then this is computed from the data.
`where`	The chromosome of the subset of data to deconvolve. Inactive (`NA`) for ChIP-seq data.
`min.npeaks`	Optionally limit the minimum number of coefficients. Default is `0` - no lower limit.
`max.npeaks`	Optionally limit the maximum number of coefficients. Default is `99999` - no upper limit.
`...`	All the other parameters of original `chip.deconv` from `MeDiChI` package such as: `boot.sample.opt = "residual"`, `max.peak = NA`, `boot.vary.res = F`, `tile.distance = NA`, `where = NA`, `min.npeaks = 0`, `max.npeaks = 99999` and `smooth=FALSE` can be also changed. Nevertheless in `deconv.entire.genome.seq` and `fit.peak.profile.seq` they are set up to the default values.

Details

See details of `chip.deconv` from `MeDiChI` package.

Author(s)

Malgorzata Nowicka <gosia.nowicka@uzh.ch> and Wouter Van Gool under the direction of Marco Antonio Mendoza Parra <marco@igbmc.fr> and Hinrich Gronemeyer

References

http://archive.igbmc.fr/recherche/Prog_FGC/Eq_HGron/bioinfotools.html

Mendoza-Parra MA, Walia M, Sankar M, Gronemeyer H. Dissecting the retinoid-induced differentiation of F9 embryonal stem cells by integrative genomics. Mol Syst Biol. 2011 Oct 11;7:538. doi: 10.1038/msb.2011.73. PubMed PMID: 21988834; PubMed Central PMCID: PMC3261707. URL: http://www.nature.com/msb/journal/v7/n1/full/msb201173.html

Mendoza-Parra MA, Sankar M, Walia M, Gronemeyer H. POLYPHEMUS: R package for comparative analysis of RNA polymerase II ChIP-seq profiles by non-linear normalization. Nucleic Acids Res. 2012 Feb;40(4):e30. doi: 10.1093/nar/gkr1205. Epub 2011 Dec 7. PubMed PMID: 22156059; PubMed Central PMCID: PMC3287170. URL: http://nar.oxfordjournals.org/content/40/4/e30.long

David J. Reiss, Marc T. Facciotti, and Nitin S. Baliga. Model-based deconvolution of genome-wide DNA binding. Bioinformatics, pages btm592+, 2007. doi: 10.1093/bioinformatics/btm592. URL: http://bioinformatics.oxfordjournals.org/cgi/content/abstract/btm592v1

See Also

`deconv.entire.genome.seq`, `fit.peak.profile.seq`, `chip.deconv`

Examples

```
# one window deconvolution for CTCF

dir <- system.file("extdata/CTCF", package="MeDiChISeq")
data <- read.table(file.path(dir,
"chr19_IP_GSM646314_GM12878_CTCF_rep1_rcd_res-10_dist-150_both.wig"), skip=2)
kernel <- read.table(file.path(dir, "MeDiChISeq_CTCF_kernel.txt"))

out <- chip.deconv.seq(data = data, center = 461582, window = 20000,
kernel = kernel, quant.cutoff = 10, fit.res = 50)

coef(out)

plot(out)


# one window deconvolution for H3K4me3

dir <- system.file("extdata/H3K4me3", package="MeDiChISeq")
data <- read.table(file.path(dir,
"chr19_IP_GSM646424_Huvec_H3K4me3_rep1_rcd_res-10_dist-150_both.wig"), skip=2)
kernel <- read.table(file.path(dir, "MeDiChISeq_H3K4me3_kernel.txt"))

out <- chip.deconv.seq(data = data, center = 469384, window = 50000,
kernel = kernel, quant.cutoff = 10, fit.res = 50)

coef(out)

plot(out)

plot(out, center = 483241, window = 5000)
```

`deconv.entire.genome.seq` *High-resolution model-based deconvolution of normalized ChIP-Seq data*

Description

Deconvolves an entire ChIP-Seq data set, across all chromosomes by running adjusted version of `chip.deconv` function from `MeDiChI` package on multiple, contiguous, overlapping subsets of the data and combining the results.

Usage

```
deconv.entire.genome.seq(file.IP, file.Control=NULL,
format="bed", genome="hg19", output.dir=NULL, output.name=NULL,
chrom.list=NULL, limL=0, limU=Inf, potential.peaks=NULL,
reads.elong=150, kernel, frag.length,
quant.cutoff="q1e-5", window=20000, wig.res=10, fit.res=50,
max.steps=100, selection.method="bic", post.proc.factor=2,
nr.boots=5, local.windows=c(1000, 2000, 5000),
Control.corr.param=0.01, nr.cores=1,
```

```
remove.clonal.reads=TRUE, clonal.reads.to.keep=3,
verbose.console=TRUE, overwrite.wigs=FALSE, keep.wigs=TRUE, ...)
```

Arguments

- `file.IP` — Path to the ChIP-Seq file.
- `file.Control` — Path to the Control file.
- `format` — Format of the ChIP-Seq file. By default "bed". Other accepted formats: "sam", "bam", "bowtie", "soap".
- `genome` — Chatacter specifying the genome "hg18", "hg19", "mm8", "mm9", "dm2", "dm3", "ce4", "ce6", "rn3", "rn4", "danRer4", "danRer6".
- `output.dir` — Directory to the folder where all the output should be saved. If such a path does not exist, it will be created. By default `output.dir = NULL` and a folder named "MeDiChISeq_output" is created in the current working directory.
- `output.name` — Character. Experiment name used when output files are generated. By default `output.name = NULL`.
- `chrom.list` — Character vector of chromosomes to be deconvolved. If `chrom.list = NULL`, whole genome is deconvolved.
- `limL` — Lowest boundary of the region to deconvolve, by default `limL = 0`
- `limU` — Upper boundary of the region to deconvolve, by default `limU = Inf`. Default values for `limL` and `limU` result in deconvolving entire chromosomes.
- `potential.peaks` — Path or `data.frame` that should have columns "chromosome", "start", "end". This is a list of regions in which potential peaks can be located.
- `reads.elong` — Path or numeric. Reads are elongated by this value in order to create intensity wig files. Should correspond to the length of the sonicated DNA fragments and can be estimated with `fit.peak.profile.seq` function. Should be the same value which was used for reads elongation when kernel was generated.
- `kernel` — Path, `data.frame` or `matrix` which is one of the outputs of `fit.peak.profile.seq`, describing the peak shape.
- `frag.length` — Path or numeric value which is one of the outputs of `fit.peak.profile.seq`. This value is used to calculate the base of annotated peaks.
- `quant.cutoff` — Intensity threshold that defines the regions of potential peaks. By assigning a numeric value it will be constant for a whole genome, when defined as quantile the threshold for each window will be calculated based on a Poisson approach. Default value `quant.cutoff = "q1e-5"` corresponds to 1 - 1e-5 = 0.99999th quantile from Poisson distribution with lambda equal to the average intensity in that window, as if it was equally distributed.
- `window` — Numeric in base pairs. Whole genome is divided into overlapping windows of this size in which the deconvolution takes place. When `quant.cutoff` is defined as quantile, the Poisson background is estimated based on the information from such windows. By default `window=20000`. It is suggested to use bigger window for broader profiles. See vignette.
- `wig.res` — Resolution of the wigs that are created.
- `fit.res` — Numeric in base pairs. Desired deconvolution resolution.
- `max.steps` — Limit the number of LARS steps taken.

selection.method
: In LARS regression the accurate model is chosen based on AIC or BIC criterion. Default is "bic".

post.proc.factor
: Post-processing filter for combining deconvolution coefficients. When equal 2, means that all the coefficients that are separated by maximum 2*fit.res will be merged. When equal 0, no merging is performed, but usually this results in profile overfitting. The value post.proc.factor * fit.res can be interpreted as minimal allowed distance between two neighbor peaks.

nr.boots
: Number of bootstrap iterations to perform. If nr.boots = 1, no bootstraps are performed and no p-values will be generated. Suggested value nr.boots = 5.

local.windows
: Numeric vector specifying the size of windows used for local assessment of confidence of putative peaks. By default local.windows = c(1000, 2000, 5000).

Control.corr.param
: Numeric value from [0,1] interval. If Control sample is available this value is the percentage of overlap that is necessary to conclude that two peaks from IP and Control are located in the same position. Consequently the confidence of IP peak is reduced.

nr.cores
: Numeric. The number of cores to be used for parallel processing. This argument is passed directly to the mclapply function.

remove.clonal.reads
: Logical. Whether the clonal reads should be removed before creating WIG files.

clonal.reads.to.keep
: Numeric. Number of reads that are accepted to have exactly the same position. Additional reads are removed.

verbose.console
: Logical. If verbose.console = TRUE, than the progress of fitting is displayed in console and saved in output.dir.

overwrite.wigs
: Logical. Whether to overwrite WIG files with the existing ones. When FALSE one can save time on WIG construction.

keep.wigs
: Logical. Whether to keep or delete the generated WIG files.

...

Value

Generated output consists of the following object:

- All.coeffs.IP that contains a list of all annotated peaks with their intensities, global, local and combined p-values and coefficients after control correction,

This object is saved in output.dir. Moreover you can find there a BED file with annotated peaks and their intensities and WIG files which can be loaded to any genome browser for visual verification of obtained results. In order to facilitate the choice of p-values cutoff a plot of intensities versus p-values is generated. The console output of deconv.entire.genome.seq is also saved.

Author(s)

Malgorzata Nowicka <gosia.nowicka@uzh.ch> and Wouter Van Gool under the direction of Marco Antonio Mendoza Parra <marco@igbmc.fr> and Hinrich Gronemeyer

References

http://archive.igbmc.fr/recherche/Prog_FGC/Eq_HGron/bioinfotools.html

Mendoza-Parra MA, Walia M, Sankar M, Gronemeyer H. Dissecting the retinoid-induced differentiation of F9 embryonal stem cells by integrative genomics. Mol Syst Biol. 2011 Oct 11;7:538. doi: 10.1038/msb.2011.73. PubMed PMID: 21988834; PubMed Central PMCID: PMC3261707. URL: http://www.nature.com/msb/journal/v7/n1/full/msb201173.html

Mendoza-Parra MA, Sankar M, Walia M, Gronemeyer H. POLYPHEMUS: R package for comparative analysis of RNA polymerase II ChIP-seq profiles by non-linear normalization. Nucleic Acids Res. 2012 Feb;40(4):e30. doi: 10.1093/nar/gkr1205. Epub 2011 Dec 7. PubMed PMID: 22156059; PubMed Central PMCID: PMC3287170. URL: http://nar.oxfordjournals.org/content/40/4/e30.long

David J. Reiss, Marc T. Facciotti, and Nitin S. Baliga. Model-based deconvolution of genome-wide DNA binding. Bioinformatics, pages btm592+, 2007. doi: 10.1093/bioinformatics/btm592. URL: http://bioinformatics.oxfordjournals.org/cgi/content/abstract/btm592v1

See Also

chip.deconv.seq, fit.peak.profile.seq

Examples

```
# if possible increase the number of cores used for parallelization
nr.cores=2
# deconvolve a small chunk of chromosome 19 from CTCF profile

dir <- system.file("extdata/CTCF", package="MeDiChISeq")
file.IP <- file.path(dir, "GSM646314_GM12878_CTCF_rep1_rcd.bed")
kernel <- file.path(dir, "MeDiChISeq_CTCF_kernel.txt")
reads.elong <- file.path(dir, "MeDiChISeq_CTCF_reads_elong.txt")
frag.length <- file.path(dir, "MeDiChISeq_CTCF_frag_length.txt")

CTCF.deconv <- deconv.entire.genome.seq(file.IP,
output.name="CTCF", genome="hg18",
quant.cutoff="q1e-5", window=20000,
chrom.list="chr19", limL=0, limU=500000,
reads.elong=reads.elong, kernel=kernel, frag.length=frag.length,
nr.cores=nr.cores, nr.boots=2,
remove.clonal.reads=FALSE)
```

---

fit.peak.profile.seq *Learn deconvolution kernel - peak shape - from ChIP-Seq data.*

Description

Learn the peak shape, to be further used for the deconvolution via deconv.entire.genome.seq.

Usage

```
fit.peak.profile.seq(file.IP, format="bed", genome="hg19", output.dir=NULL,
output.name=NULL, chrom.fit=NULL, limL=0, limU=Inf,
reads.elong=150, quant.cutoff="q1e-7", window=20000, mini.window=2000,
wig.res=10, fit.res=50, reads.length=50,
n.peaks = 50, n.skip = 20, re.fit=100, max.iter=500,
selection.method="bic", post.proc.factor=2,
start.pars =c(shape =10, scale = 20), to.be.fit=c("shape", "scale"),
method = "Nelder-Mead", nr.cores=1,
remove.clonal.reads=TRUE, clonal.reads.to.keep=3,
write.pdf=TRUE, save.kernel=TRUE, verbose.console=TRUE,
overwrite.wigs=FALSE, keep.wigs=TRUE, ...)
```

Arguments

`file.IP`	Path to the ChIP-Seq file.
`format`	Format of the ChIP-Seq file. By default `"bed"`. Other accepted formats are `"sam"`, `"bam"`, `"bowtie"`, `"soap"`.
`genome`	Chatacter specifying the genome `"hg18"`, `"hg19"`, `"mm8"`, `"mm9"`, `"dm2"`, `"dm3"`, `"ce4"`, `"ce6"`, `"rn3"`, `"rn4"`, `"danRer4"`, `"danRer6"`.
`output.dir`	Directory to the folder where all the output should be saved. If such a path does not exist, it will be created. By default `output.dir = NULL` and a folder named "MeDiChISeq_output" is created in the current working directory.
`output.name`	Character. Experiment name used when output files are generated. By default `output.name = NULL`.
`chrom.fit`	Character specifying the chromosome on which the learning should be performed. If `fit.chrom = NULL`, the shortest chromosome is taken.
`limL`	Lowest boundary of the region to deconvolve, by default `limL = 0`
`limU`	Upper boundary of the region to deconvolve, by default `limU = Inf`. Default values for `limL` and `limU` result in entire chromosomes deconvolution.
`reads.elong`	Numeric. Reads are elongated by this value in order to create intensity WIG files. Should correspond to the average length of sonicated DNA fragments. When `reads.elong = NULL`, average fragment length is estimated based on forward and reverse profiles. See vignette.
`quant.cutoff`	Intensity threshold that defines the regions of potential peaks. By assigning a numeric value it will be constant for a whole genome, when defined as quantile the threshold for each window will be calculated based on Poisson approach. Default value `quant.cutoff = "q1e-7"` corresponds to 1 - 1e-7 = 0.9999999th quantile from Poisson distribution with lambda equal to the average intensity in that window, as if it was equally distributed.
`window`	Numeric in base pairs. Whole genome is divided into overlapping windows of this size in which the deconvolution takes place. When `quant.cutoff` is defined as quantile, the Poisson background is estimated based on the information from such windows. By default `window=20000`. It is sugested to use bigger window for broader profiles. See vignette.
`mini.window`	Numeric in base pairs. Size of the window to be plotted around each of the bright peaks.
`wig.res`	Resolution of the wigs that are created.

`fit.res`	Numeric in base pairs. Desired deconvolution resolution.
`reads.length`	Length of alligned reads. This value is used during the estimation of `reads.elong`. See vignette.
`n.peaks`	Number of regiones with biggest peaks to learn from.
`n.skip`	Number of regiones with worst-fitting to skip. Enables filtering out of peaks that do not agree with the majority of isolated peaks.
`re.fit`	Re-run `deconv.entire.genome.adj` on input data using current best-fit profile, every `re.fit` iterations.
`max.iter`	Maximum number of iterations for `"Nelder-Mead"`, `"BFGS"`, `"CG"` methods. See `optim` function.
`selection.method`	In LARS regression the accurate model is chosen based on AIC or BIC criterion. Default is `"bic"`.
`post.proc.factor`	Post-processing filter for combining deconvolution coefficients. When equal 2, means that all the coefficients that are separated by maximum 2*`fit.res` will be merged. When equal 0, no merging is performed, but usually this results with profile overfitting. The value `post.proc.factor` * `fit.res` can be interpreted as minimal allowed distance between two neighbor peaks.
`start.pars`	Starting parameters for model profile. By default `c(shape =10, scale = 20)`, which corresponds to the average DNA fragment length of 200bp.
`to.be.fit`	Names of parameters to be learned.
`method`	Optimization method, by default `method = "Nelder-Mead"`. See `optim` function.
`nr.cores`	Numeric. The number of cores to be used for parallel processing. This argument is passed directly to the `mclapply` function.
`remove.clonal.reads`	Logical. If the clonal reads should be removed before creating WIG files.
`clonal.reads.to.keep`	Numeric. Number of reads that are accepted to have exactly the same position. Additional reads are removed.
`write.pdf`	Logical. Whether pdf plots of fitted kernels should be generated and saved in the `output.dir`.
`save.kernel`	Logical. Whether the files with `kernel` and `frag.length` should be saved in the `output.dir`.
`verbose.console`	Logical. If `verbose.console = TRUE`, than the progress of fitting is displayed in console and saved in `output.dir`.
`overwrite.wigs`	Logical. Whether to overwrite WIG files with the existing ones. When `FALSE` one can save time on WIGs construction.
`keep.wigs`	Logical. Whether to keep or delete the generated WIG files.
`...`	

Value

A list of 3 objects:

- `reads.elong`: see above.

- kernel: data.frame with the final peak profile.
- frag.length: estimated fragment length. Base of the peak profile is assumed to be equal to 2*frag.length.

Moreover, if kernel=TRUE, verbose.console=TRUE, keep.wigs=TRUE, than in the directory output.dir all the above outputs will be saved together with generated WIG files, console display of work progress and the PDF files with kernel plots.

Author(s)

Malgorzata Nowicka <gosia.nowicka@uzh.ch> and Wouter Van Gool under the direction of Marco Antonio Mendoza Parra <marco@igbmc.fr> and Hinrich Gronemeyer

References

http://archive.igbmc.fr/recherche/Prog_FGC/Eq_HGron/bioinfotools.html

Mendoza-Parra MA, Walia M, Sankar M, Gronemeyer H. Dissecting the retinoid-induced differentiation of F9 embryonal stem cells by integrative genomics. Mol Syst Biol. 2011 Oct 11;7:538. doi: 10.1038/msb.2011.73. PubMed PMID: 21988834; PubMed Central PMCID: PMC3261707. URL: http://www.nature.com/msb/journal/v7/n1/full/msb201173.html

Mendoza-Parra MA, Sankar M, Walia M, Gronemeyer H. POLYPHEMUS: R package for comparative analysis of RNA polymerase II ChIP-seq profiles by non-linear normalization. Nucleic Acids Res. 2012 Feb;40(4):e30. doi: 10.1093/nar/gkr1205. Epub 2011 Dec 7. PubMed PMID: 22156059; PubMed Central PMCID: PMC3287170. URL: http://nar.oxfordjournals.org/content/40/4/e30.long

David J. Reiss, Marc T. Facciotti, and Nitin S. Baliga. Model-based deconvolution of genome-wide DNA binding. Bioinformatics, pages btm592+, 2007. doi: 10.1093/bioinformatics/btm592. URL: http://bioinformatics.oxfordjournals.org/cgi/content/abstract/btm592v1

See Also

chip.deconv.seq, deconv.entire.genome.seq

Examples

```
# if possible increase the number of cores used for parallelization
nr.cores=2

# finds kernel for CTCF profile

dir <- system.file("extdata/CTCF", package="MeDiChISeq")
file.IP <- file.path(dir, "GSM646314_GM12878_CTCF_rep1_rcd.bed")

CTCF.peak.profile <- fit.peak.profile.seq(file.IP, genome="hg18", chrom.fit="chr19",
output.name="CTCF", window=20000, mini.window=2000, quant.cutoff=20,
limL=0, limU=1000000, re.fit=15, max.iter=20,
start.pars =c(shape = 17, scale = 10), nr.cores=nr.cores,
remove.clonal.reads=FALSE)
```

`remove.clons` *Remove clonal reads*

Description

Removes reads that starts in exactly the same position.

Usage

```
remove.clons(file, reads.to.keep, output.dir, format)
```

Arguments

`file`	Path to the file containing alligned reads.
`reads.to.keep`	Numeric. Defines number of reads that are allowed to have exactly the same position.
`output.dir`	Directory to the folder where new BED file will be saved.
`format`	Format of the ChIP-Seq file. Accepted formats: `"sam"`, `"bam"`, `"bowtie"`, `"soap"`.

Details

It can happen that profile will have a lot of reads starting in exactly the same position. Such reads are usually considered as artefacs resulting from "clonal" amplification during the PCR phase in ChIP-seq experiment. If this is a frequent phenomena they can have an influence on the shape of peaks. Such peaks follow a square pattern. That is why we suggest to remove so called clonal reads.

Value

Produces new cleaned BED file in pointed directory. Output of `remove.clons` a path to the generated BED file.

Author(s)

Malgorzata Nowicka <gosia.nowicka@uzh.ch> and Wouter Van Gool under the direction of Marco Antonio Mendoza Parra <marco@igbmc.fr> and Hinrich Gronemeyer

Examples

```
dir <- system.file("extdata/CTCF", package="MeDiChISeq")
file <- file.path(dir, "GSM646314_GM12878_CTCF_rep1_rcd.bed")

remove.clons(file, reads.to.keep=2, output.dir=getwd(), format="bed")
```

`write.wigs.parallel` *WIG generator*

Description

Produces WIG files from files containing aligned reads.

Usage

```
write.wigs.parallel(file, output.dir, chromosomes, sample.type,
wig.res, reads.elong, split = FALSE, genome, format, zeros = F,
nr.cores = 1, overwrite.wigs = FALSE, verbose = T)
```

Arguments

`file`	Path to the file containing aligned reads.
`output.dir`	Directory to the folder where all the WIGS will be saved.
`chromosomes`	List of chromosomes for which the WIGS will be produced.
`sample.type`	Name prefix of the new WIG files.
`wig.res`	Resolution of the wigs.
`reads.elong`	Numeric. Reads are elongated by this value in order to create intensity wig files.
`split`	Logical. If `TRUE`, separet wigs for forward and reverse strand will be produced.
`genome`	Chatacter specifying the genome `"hg18"`, `"hg19"`, `"mm8"`, `"mm9"`, `"dm2"`, `"dm3"`, `"ce4"`, `"ce6"`, `"rn3"`, `"rn4"`, `"danRer4"`, `"danRer6"`.
`format`	Format of the ChIP-Seq file. Accepted formats: `"sam"`, `"bam"`, `"bowtie"`, `"soap"`.
`zeros`	Logical. Whether to produce the entries with intensity zero.
`nr.cores`	Numeric. The number of cores to be used for parallel processing.
`overwrite.wigs`	Logical. Whether to overwrite wig files with the existing ones. When `FALSE` one can save time on wig construction.
`verbose`	Logical. Whether to display processing status.

Value

Produces the WIG files in pointed directory. Output of `write.wigs.parallel` is a list of paths to the generated WIGS.

Author(s)

Malgorzata Nowicka <gosia.nowicka@uzh.ch> and Wouter Van Gool under the direction of Marco Antonio Mendoza Parra <marco@igbmc.fr> and Hinrich Gronemeyer

Examples

```
dir <- system.file("extdata/CTCF", package="MeDiChISeq")
file <- file.path(dir, "GSM646314_GM12878_CTCF_rep1_rcd.bed")

write.wigs.parallel(file, output.dir=getwd(), chromosomes="chr19", sample.type="IP",
wig.res=10, reads.elong=150, split = FALSE, genome="hg18", format="bed", zeros = FALSE,
nr.cores = 1, overwrite.wigs = FALSE, verbose = TRUE)
```

Index

chip.deconv.seq, 2, *7*, *10*
coef.chip.deconv.seq *(*chip.deconv.seq*)*, 2

deconv.entire.genome.seq, *2, 3*, 4, *10*

fit.peak.profile.seq, *2, 3*, *5*, *7*, 7

mclapply, *6*, *9*

optim, *9*

plot.chip.deconv.seq *(*chip.deconv.seq*)*, 2
print.chip.deconv.seq *(*chip.deconv.seq*)*, 2

remove.clons, 11

write.wigs.parallel, 12
