# Supplementary material for: Characterising ChIP-seq binding patterns by model-based peak shape deconvolution
Source: BMC Genomics. 2013 Nov 26;14(1):834. doi: 10.1186/1471-2164-14-834 (PMC4046686; doi:10.1186/1471-2164-14-834)
Supplement: Supplementary file 7 — Additional file 7: Peak caller characteristics and their relevant parameters used in this study. (PDF 7 KB) [file 12864_2013_5524_MOESM7_ESM.pdf]

Peak caller	version	Peak identification strategy	Read elongation distance prediction	Input background subtraction	Relevant parameters in use
**MACS**	1.4	local Poisson distribution	Strand-specific shifting model	when available	p-value $<1x10^{-5}$
**BayesPeak**	1.10.0	Signal enrichment dependency (Hidden Markov model and Bayesian statistical methodology)	none	when available	Posterior probability PP>0.5
**PeakRanger**	1.16	Summit-valley alternator	Reads elongation; -l 150	mandatory	p-value $<1X10^{-4}$ FDR<0.01
**MeDiChISeq**	1.0.8	Model-based deconvolution	Reads elongation; reads.elong=150	when available	Intensity threshold quant.cutoff≥10

Additional file 4. Peak caller characteristics and their relevant parameters used in this study.
