# Supplementary material for: Characterising ChIP-seq binding patterns by model-based peak shape deconvolution
Source: BMC Genomics. 2013 Nov 26;14(1):834. doi: 10.1186/1471-2164-14-834 (PMC4046686; doi:10.1186/1471-2164-14-834)
Supplement: Supplementary file 8 — Additional file 8: Overlapping peaks identified by different peak calling algorithms. (PDF 16 KB) [file 12864_2013_5524_MOESM8_ESM.pdf]

NRSF

Replicate	Method (total sites)	MeDiChISeq	MACS	BayesPeak	PeakRanger
Replicate 1	MeDiChISeq (16,429)		30	46	30
	MACS (5,843)	85		71	72
	BayesPeak (78,558)	10	5		5
	PeakRanger (5,165)	97	82	74	
Replicate 2	MeDiChISeq (52,582)		12	37	14
	MACS (7,005)	88		70	73
	BayesPeak (109,665)	18	4		5
	PeakRanger (7,894)	93	65	68	

SRF

Replicate	Method (total sites)	MeDiChISeq	MACS	BayesPeak	PeakRanger
Replicate 1	MeDiChISeq (13,260)		18	45	10
	MACS (5,522)	44		75	23
	BayesPeak (113,328)	5	4		1
	PeakRanger (1,404)	93	92	70	
Replicate 2	MeDiChISeq (15,277)		22	52	10
	MACS (8,386)	40		79	19
	BayesPeak (150,453)	5	4		1
	PeakRanger (1,650)	97	95	75	

MAX

Replicate	Method (total sites)	MeDiChISeq	MACS	BayesPeak	PeakRanger
Replicate 1	MeDiChISeq (13,029)		79	66	39
	MACS (28,706)	36		74	18
	BayesPeak (63,903)	13	33		5
	PeakRanger (5,359)	94	95	66	
Replicate 2	MeDiChISeq (7,755)		84	66	41
	MACS (16,802)	39		71	19
	BayesPeak (44,719)	11	27		5
	PeakRanger (3,409)	94	95	64	

H3K9ac

Replicate	Method (total sites)	MeDiChISeq	MACS	BayesPeak	PeakRanger
Replicate 1	MeDiChISeq (67,402)		26	32	36
	MACS (23,317)	77		43	59
	BayesPeak (48,199)	44	21		20
	PeakRanger (27,274)	88	51	36	
Replicate 2	MeDiChISeq (45,104)		28	24	32
	MACS (20,684)	62		45	45
	BayesPeak (45,179)	23	21		10
	PeakRanger (15,570)	92	60	30	

H3K27ac

Replicate	Method (total sites)	MeDiChISeq	MACS	BayesPeak	PeakRanger
Replicate 1	MeDiChISeq (103,092)		22	27	27
	MACS (35,905)	63		48	59
	BayesPeak (78,405)	35	22		18
	PeakRanger (37,139)	76	57	38	
Replicate 2	MeDiChISeq (84,189)		25	23	27
	MACS (35,829)	59		49	44
	BayesPeak (67,987)	29	26		12
	PeakRanger (24,830)	92	63	34	

Peaks overlap (%)
50 100

Additional file 5. Fraction of commonly identified peaks by different peak calling algorithms. Binding sites identified by different peak callers were compared in the context of their summit locations ± 50nt. The fraction of overlapping peaks (in percentage) relative to the total sites for a given method (indicated at the right side of each panel) is displayed and illustrated as heatmap. Note that in most cases MeDiChISeq presents the higher fraction of overlapping peaks when compared with other peak callers.
