# Supplementary material for: Characterising ChIP-seq binding patterns by model-based peak shape deconvolution
Source: BMC Genomics. 2013 Nov 26;14(1):834. doi: 10.1186/1471-2164-14-834 (PMC4046686; doi:10.1186/1471-2164-14-834)
Supplement: Supplementary file 9 — Additional file 9: MeDiChISeq binding sites identification at different sequencing depth levels compared with that performed by other peak caller approaches. (PDF 187 KB) [file 12864_2013_5524_MOESM9_ESM.pdf]

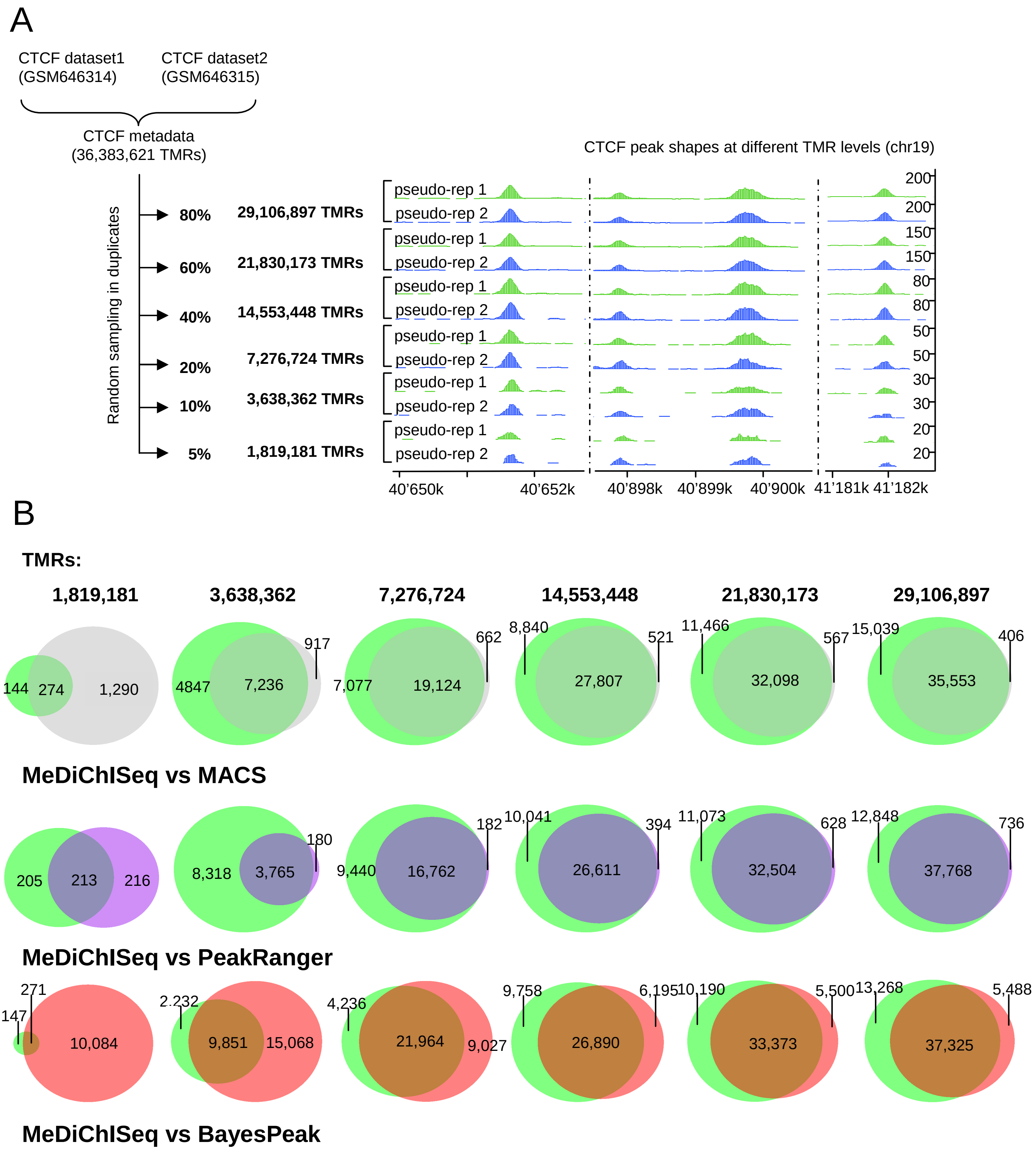

Additional file 6. Assessing the influence of the sequencing depth on the performance of compared peak callers. (A) Two CTCF ChIP-seq datasets were merged to create a *meta* dataset of more than 36M reads; then subsets were derived by random sampling at indicated levels; each sub-sampling was done twice independently to obtain pseudo replicates. **(B)** Venn-diagrams illustrating the number of binding sites identified by different peak callers relative to MeDiChISeq for CTCF subset profiles with different total mapped reads (TMRs).
