# Supplementary material for: Characterising ChIP-seq binding patterns by model-based peak shape deconvolution
Source: BMC Genomics. 2013 Nov 26;14(1):834. doi: 10.1186/1471-2164-14-834 (PMC4046686; doi:10.1186/1471-2164-14-834)
Supplement: Supplementary file 10 — Additional file 10: Detailed analysis of the binding sites identification performed by MeDiChISeq and MACS in a CTCF ChIP-seq profile reconstructed from 29’100,000 TMRs. (PDF 109 KB) [file 12864_2013_5524_MOESM10_ESM.pdf]

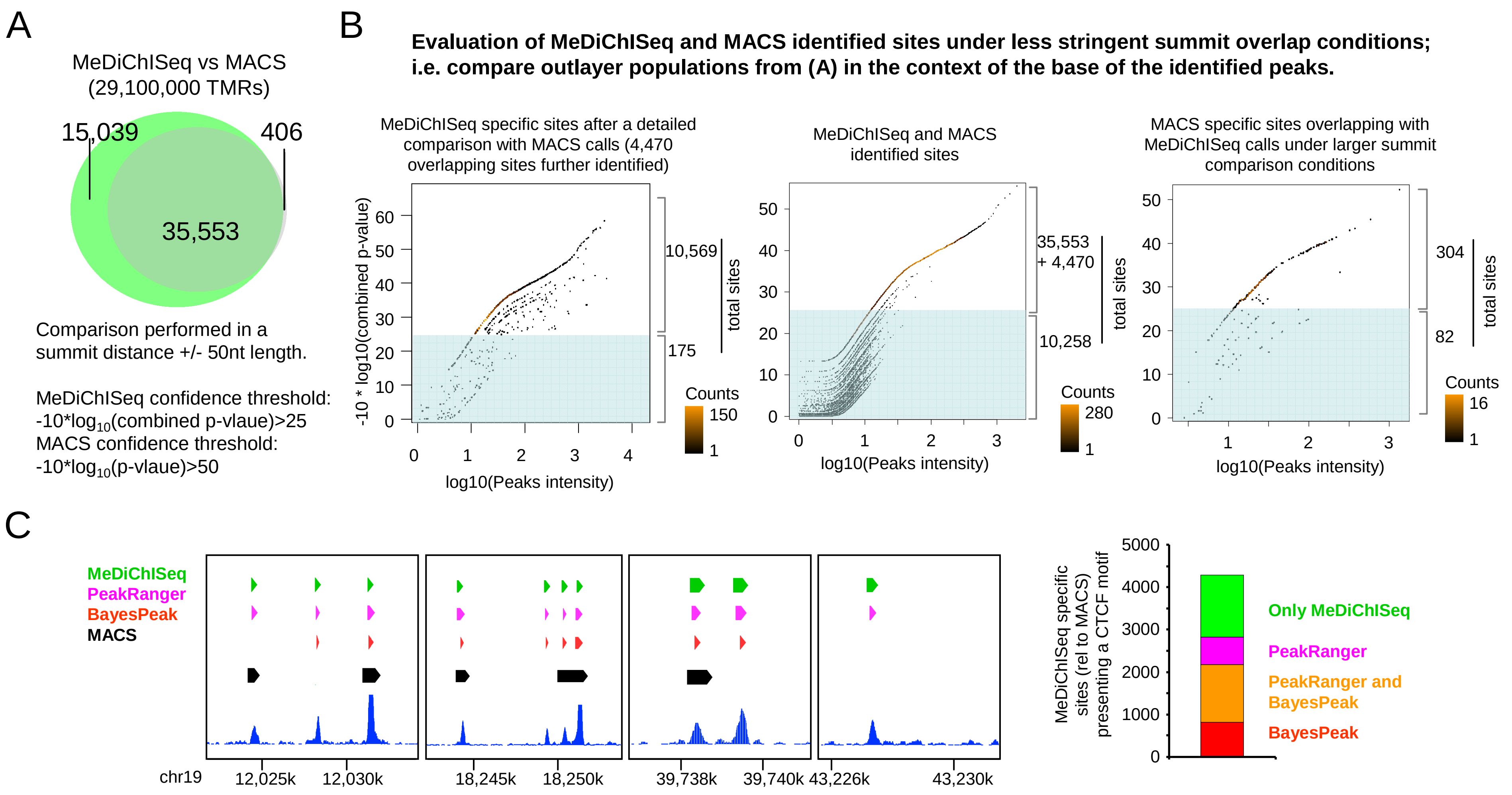

Additional file 7. Evaluation of MeDiChISeq-specific sites retrieved in the CTCF *meta* data (80% total mapped read subset). (A) MeDiChISeq retrieved most of the sites annotated by MACS plus additional 15,000 specific sites. **(B)** When more relaxed conditions are used for assessing peak overlaps (i.e., base peak overlaps) 4,470 (MeDiChISeq-specific for stringent conditions) + 406 (MACS-specific for stringent conditions) additional overlapping MACS-MeDiChISeq sites were identified. The remaining 10,744 MeDiChISeq-specific sites are illustrated in the context of their intensity and confidence (left panel) to show that they present significant binding site characteristics (only 175 sites with confidence levels below 25 [light blue area]) comparable to that observed for the overlapping sites (middle and right panel). **(C)** 4,291 MeDiChISeq-specific sites out of 10,569 present a CTCF motif (CentriMo: p-value: $4.4x10^{-1085}$; see **Figure 4**). Some of these sites are also identified by PeakRanger and/or BayesPeak supporting the fact that several sites which are not retrieved by MACS correspond to real binding events.
