# Supplementary material for: Characterising ChIP-seq binding patterns by model-based peak shape deconvolution
Source: BMC Genomics. 2013 Nov 26;14(1):834. doi: 10.1186/1471-2164-14-834 (PMC4046686; doi:10.1186/1471-2164-14-834)
Supplement: Supplementary file 11 — Additional file 11: H3K36me3 profiling analyzed by MeDiChISeq and compared with the cluster identification approach SICER. (PDF 24 KB) [file 12864_2013_5524_MOESM11_ESM.pdf]

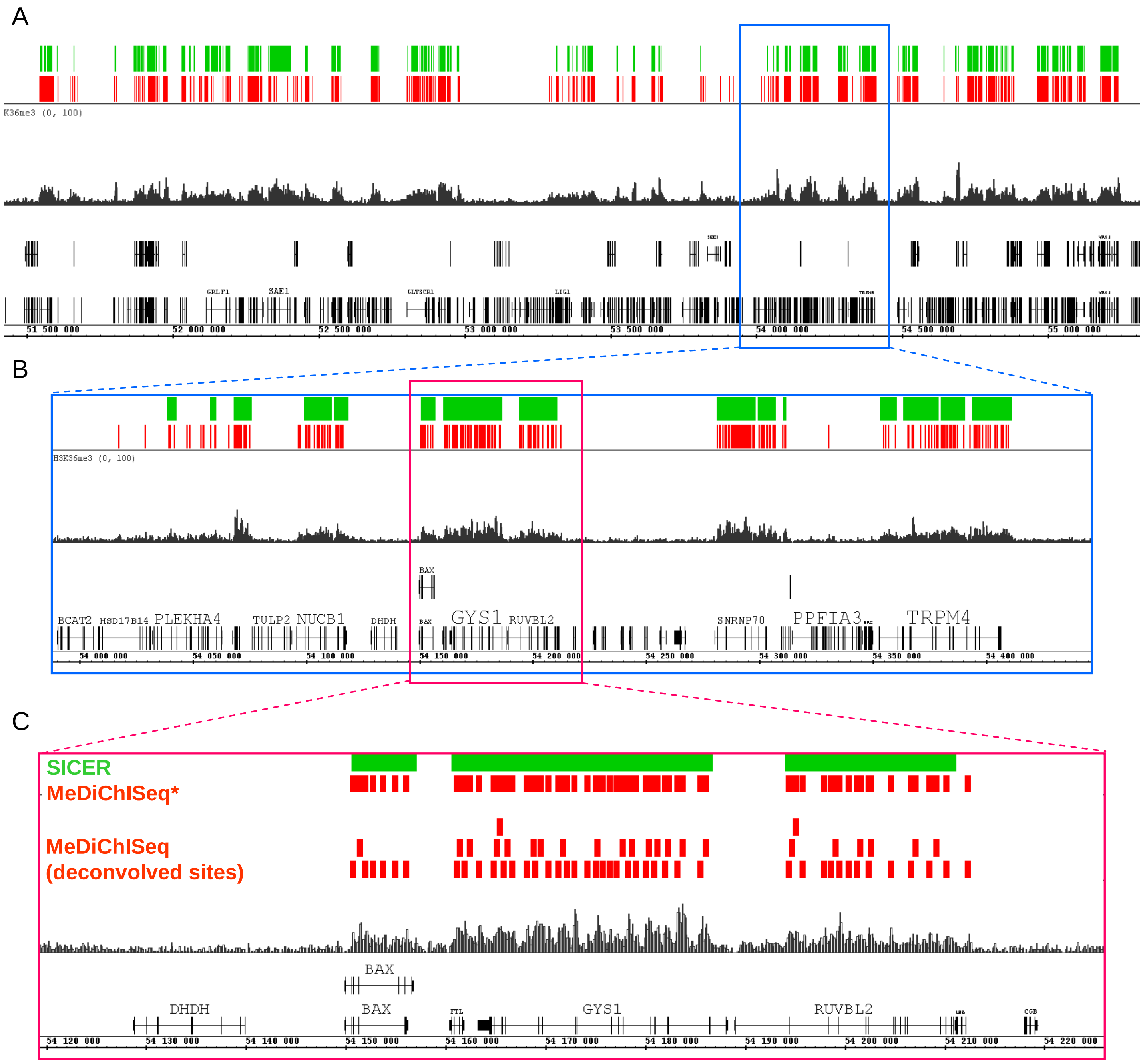

Additional file 8. H3K36me3 profiling analyzed by MeDiChISeq and compared with the clustering identification approach, SICER (*C. Zang et al; Bioinformatics 25, 15:1952-1958; 2009*). For comparative purposes, MeDiChISeq overlapping sites were merged when available as displayed in (A); (B) and (C). In (C) the location of deconvolved peaks prior to merging is also displayed to illustrate the degree of resolution obtained with MeDiChISeq. This example shows that, while MeDiChISeq is not an "enrichment island" identificator, it can accurately identify emrichment clustering regions as highlighted with the comparison with SICER islands predictions.
